# A Novel Approach for the Activity Assessment of L-Asparaginase Formulations When Dealing with Complex Biological Samples

**DOI:** 10.3390/ijms26115227

**Published:** 2025-05-29

**Authors:** Igor D. Zlotnikov, Elena V. Kudryashova

**Affiliations:** Faculty of Chemistry, Lomonosov Moscow State University, Leninskie Gory, 1/3, 119991 Moscow, Russia; zlotnikovid@my.msu.ru

**Keywords:** L-asparaginase, acute lymphoblastic leukemia, fluorometric assay, complex biological samples, therapeutic formulations

## Abstract

Majority of commercial L-asparaginase (L-ASNase) activity assays are based on coupled enzymatic reaction, which converts aspartate into pyruvate, subsequently reacting with the probe to form a stable chromophore, which can be detected spectrophotometrically. However, in complex biological samples this method can be inaccurate due to poor optical transparency or presence of compounds interfering with the coupled enzyme reaction–for this kind of cases alternative methods have been suggested. Here we suggest a strategy to rationally pick a method of choice in a variety of situations, taking into consideration the upsides and downsides of each method. A high-throughput fluorometric assay employing the substrate Asp-AMC was rigorously validated for L-ASPNase activity screening. Aassay performance is evaluated in complex biological matrices, including bovine serum, whole and diluted human blood, and finally the mouse blood and liver homogenates samples obtained from pharmacokinetic studies. This comprehensive validation process ensures the reliability and applicability of the assay for assessing L-asparaginase activity in diverse and physiologically relevant environments. Potential interfering factors and matrix effects were addressed, and assay conditions were optimized for each matrix. The optimized assay was employed to screen various L-asparaginase types (intracellular L-ASNases type I RrA, periplasmic L-ASNases type II EcA and EwA) and ASPNase formulations (conjugates with polyamines or polyelectrolyte complexes), comparing their kinetic parameters and stability. Fourier-transform infrared (FTIR) spectroscopy was further employed to investigate the fine features of molecular mechanisms of L-asparaginase catalysis. FTIR spectra of Asn during hydrolysis were analyzed in buffer solutions and in complex biological matrices, such as blood sample or liver homogenates which is crucial in the context of pharmacokinetic research. This combined fluorometric and FTIR approach provides a powerful platform for optimizing L-ASNase formulations and therapeutic strategies for ALL. Based on the results obtained we have developed a strategy to choose an approach for L-Asparaginase activity assessment for a variety of difficult situations when dealing with complex biological samples.

## 1. Introduction

Acute lymphoblastic leukemia (ALL) is the most prevalent form of cancer affecting children, constituting approximately 25–40% of childhood malignancies [1,2,3]. L-Asparaginase (L-ASNase) represents a pivotal enzyme in the management of ALL, particularly in pediatric patients [4,5,6]. The mechanism of its antineoplastic activity is based on the process of hydrolysis of L-asparagine, a crucial amino acid that is indispensable for the proliferation of leukemia cells. Unlike normal cells, leukemia cells are incapable of synthesizing asparagine on their own. The depletion of extracellular L-asparagine through L-ASNase administration leads to compromised protein synthesis and subsequent apoptosis in leukemia cells. However, the efficacy of L-ASNase treatment may be influenced by a multitude of factors, such as pharmacokinetics, degree of cellular interaction, immunogenicity, and the emergence of resistance mechanisms [7,8,9,10,11,12,13,14,15]. Therefore, accurate assessment of L-ASNase activity both in vitro and in patient blood plasma samples is essential for optimizing cancer therapy and enhancing treatment outcomes [16]. This aspect holds particular significance in the development of novel therapeutic agents and the implementation of personalized treatment strategies.

Recombinant L-ASNases from diverse bacterial sources such as *Escherichia coli* (EcA), *Erwinia chrysanthemi* (ErA), *Erwinia carotovora* (EwA), *Yersinia pseudotuberculosis* (YpA), *Rhodospirillum rubrum* (RrA), and *Wollinella succinogenes* (WsA) have been developed [17,18]. These enzymes exhibit distinct kinetic properties, varying in stability and immunogenicity. Currently, several periplasmic L-ASNase preparations with micromolar *K*_M_ and high k_cat_ values are employed in clinical practice. These include recombinant EcA, PEGylated EcA (PEG-asparaginase), and ErA. PEGylation enhances the half-life of the enzyme within the bloodstream, resulting in reduced frequency of medication administration. ErA is employed in individuals who experience allergic reactions to *E. coli* L-ASNase and PEG-asparaginase.

Furthermore, conjugates of L-ASNase with a variety of carriers, designed to enhance the pharmacokinetic characteristics and mitigate the immunogenicity of the enzyme, are of significant interest. These carriers encompass natural polycations such as oligoamines, including spermine and spermidine, as well as derivatives of cationic polymers, like chitosan, polyethyleneimine (PEI), PEG–chitosan, PEI-g-PEG, etc. [19,20,21,22]. The conjugation of L-ASNase with these copolymers can result in an extension of the enzyme’s circulation time in the bloodstream, an enhancement of its accumulation in tumor tissue, and a reduction in the body’s response to the presence of an alien protein.

Beyond the modifications discussed previously, advanced drug delivery systems like polymeric micelles and polyelectrolyte complexes offer further avenues for optimizing L-ASNase therapy. Our published work on “smart aikido” polymeric micelles demonstrates a promising approach [23]. These micelles, comprising a three-dimensional polymer matrix, encapsulate both native L-ASNase and its cationic polymer conjugates. This encapsulation strategy provides a controlled release mechanism, shielding the enzyme from proteolytic degradation and reducing its immunogenicity, thereby addressing two key limitations of current L-ASNase therapy. Notably, incorporating L-ASNase into these micellar systems significantly improves the pharmacokinetic profile, extending the enzyme’s half-life and enhancing tumor targeting. This translates to a reduced dosing frequency and potentially fewer systemic side effects.

Furthermore, polyelectrolyte complexes (PECs) [20,24,25] formed by combining L-ASNase or its cationic conjugates with anionic polymers offer another strategy for enhancing biocatalytic properties. The electrostatic interactions within these PECs can stabilize the enzyme, modulate its activity, and potentially improve its delivery to target cells. By creating a more favorable microenvironment for L-ASNase, PECs can further contribute to reducing the required dose and minimizing adverse effects.

The ongoing development of novel L-ASNase formulations extends to the exploration of engineered enzyme variants. Our recent work in the journal *Catalysts* characterized wild-type *Rhodospirillum rubrum* L-asparaginase (RrA) and three mutants with substitutions near the active site and subunit interface [26]. Indeed, the significance of electrostatic interactions within the realm of inter-unit contacts within the enzyme’s active site in terms of promoting structural stability and augmenting both catalytic and anti-proliferative functions has been elucidated.

The development of novel mutant and modified L-ASNases, as well as the enzymes from diverse sources, necessitates a robust and highly sensitive method for activity assessment. This need is particularly critical when investigating the pharmacokinetic properties of modified enzyme preparations used in in vivo experiments. Several methods exist for determining L-ASNase activity in biological fluids, each with its own advantages and limitations. Traditional methods, such as the Berthelot reaction [27] and direct Nesslerization [28], measure ammonia released upon asparagine hydrolysis. While established, these methods lack the sensitivity and adaptability needed for complex matrices like serum or blood. ELISA-based antibody assays can detect the presence of anti-asparaginase antibodies, providing insight into potential treatment resistance, but they do not directly measure enzyme activity or distinguish between neutralizing and non-neutralizing antibodies [29]. Ylikangas et al. showed that fluorimetry analysis (with Asp-AMC substrate) allowed them to determine the L-ASNase activity (5–160 µM/min) in the blood of ALL patients during the treatment [30]. Lanvers-Kaminsky et al. suggested using the L-ASNase activity assay based on the modified AHA-test with L-aspartate β-hydroxamate; however, the activity was found to be significantly lower compared to the results obtained using the MAAT method [30]. Simultaneously, the challenge of devising methods for quantifying diverse L-asparaginase (L-ASNase) preparations in human serum continues to be of paramount importance. Nonetheless, methods based on spectrophotometry present limitations in their application [31,32,33], particularly when applied to the analysis of asparaginase formulations and blood samples.

Although highly sensitive, chromatographic techniques like HPLC [34] require complex sample preparation, making them less suitable for routine clinical monitoring, especially when enzyme levels are low as in determining activities in pharmacokinetic studies. These limitations underscore the need for a more robust and streamlined assay for assessing L-ASNase activity.

A sensitive and high-throughput activity assay is of paramount importance in the investigation of the pharmacokinetic characteristics of the enzyme and its biodistribution in organs. The half-life of elimination for various forms of L-asparaginases differs: for native EcA, it is 26–30 h; for PEGylated EcA, 5.5–7 days; and for ErA, 16 h [34]. Nonetheless, following the administration of EwA, a substantial variation in serum concentrations of the patients is observed, ranging up to two orders of magnitude [35]. This finding underscores the necessity for a tailored approach to the management of leukemia, and our method offers improved sensitivity for accurate determination of pharmacokinetic parameters, such as half-life, which will be discussed further in the context of ex vivo experiments.

In this paper, we present a robust, high-throughput method for assessing L-ASNase activity in both in vitro and ex vivo systems. This method is compatible with optically opaque, multi-component media, including whole blood and serum, enabling activity determination with minimal or no sample preparation, streamlining the analysis process, and offering a significant advantage over existing techniques. This capability is crucial for evaluating the efficacy and behavior of L-ASNases in vivo. We propose a fluorometric approach for swift and precise in vitro evaluation of L-ASNase activity. Furthermore, we have devised a method based on Fourier-transform infrared (FTIR) spectroscopy to delve into the molecular mechanisms underlying L-ASNase catalysis, potentially paving the way for the rational design of novel, more potent, and less toxic enzyme formulations.

The approach suggested would significantly contribute to the optimization of antileukemic therapies and improve the prognosis for individuals with acute lymphoblastic leukemia, particularly those with recurrent or treatment-resistant forms of the disease.

## 2. Results and Discussion

### 2.1. Fluorescent High-Throughput Screening of L-ASNase Activity

#### 2.1.1. Fluorescent Properties of L-ASNase Substrate and Product

**Figure 1:**
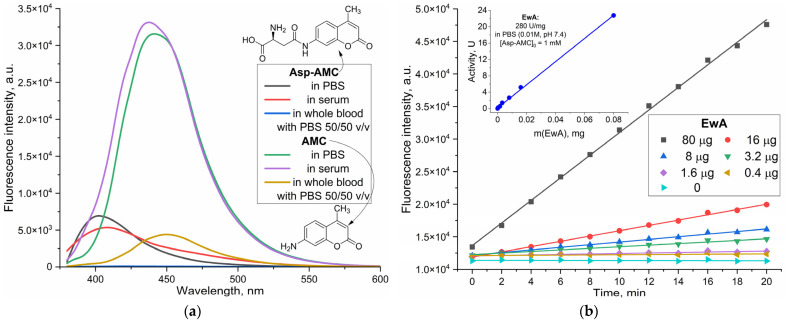
(**a**) Fluorescence emission spectra of 5 µM AMC and Asp-AMC fluorescent product and substrate for L-ASNase in PBS buffer, bovine blood serum, and whole blood diluted with PBS (50/50 *v*/*v*). λexci = 360 nm. pH = 7.4. T = 37 °C. (**b**) Example of kinetic curves of Asp-AMC hydrolysis (1 mM) under the action of L-ASNase in PBS (pH 7.4, 0.01 M). λexci = 360 nm. λemi = 460 nm. T = 37 °C. The insert shows the linear dependence of the observed activity on the amount of the enzyme.

Fluorometric assays, leveraging the difference in fluorescence properties between a substrate and its product, offer a promising approach for high-throughput screening and kinetic studies. The presented data (Figure 1) explore the applicability of a fluorometric method for L-ASNase activity determination based on the fluorescence emission spectra of the substrate, Asp-AMC, and its product, AMC (7-amino-4-methylcoumarin), in various biological systems. Figure 1a displays the emission spectra of both 5 µM AMC and Asp-AMC in phosphate-buffered saline (PBS), bovine blood serum, and whole blood diluted with PBS (50/50 *v*/*v*), providing insights into the potential impact of these complex matrices on fluorescence measurements. Furthermore, Figure 1b illustrates representative kinetic curves of Asp-AMC hydrolysis (1 mM) by L-ASNase in PBS, demonstrating the feasibility of monitoring reaction progress using this fluorogenic substrate. The insert of Figure 1b shows a graph of the linear dependence of the observed activity on the enzyme amount, where the native EwA activity is determined as 280 IU/mg.

There is a marked discrepancy in the fluorescence spectra of Asp-AMC and AMC, as depicted in Figure 1a. The maximum emission wavelength for Asp-AMC is approximately 400–405 nm, whereas for AMC, it is shifted towards the blue region, peaking at around 440–450 nm. To maximize the sensitivity and selectivity of the method, a wavelength near the maximum absorption peak of AMC at 360 nm was employed for excitation (Supplementary Figure S1), while a wavelength of 460 nm was chosen for AMC detection, to achieve the greatest fluorescence difference between substrate and product, as well as maintaining the same quantum yield of AMC fluorescence in both bovine serum and buffer. This selectivity enables direct, real-time monitoring of reaction kinetics via fluorescence enhancement, as illustrated in Figure 1b, offering a continuous and sensitive measure of L-asparaginase activity.

The fluorescence intensity of both Asp-AMC and AMC exhibits a significant variation depending on the reaction medium (Figure 1a). In blood serum, the fluorescence intensity of Asp-AMC is found to be 40% higher compared to that in PBS. Conversely, for AMC (reaction product), the maximum intensity is by 10% greater in PBS, than in serum, although it remains the same beyond a wavelength of 450 nm. Thus, by registering the signal at a wavelength of 460–480 nm, we obtain a high product/substrate ratio (>10:1) and the same quantum fluorescence yields of the product in PBS and blood serum, consequentially reducing the likelihood of error. There is a marked attenuation of fluorescence in whole blood, which significantly reduces the intensity of fluorescence for both Asp-AMC and AMC when whole blood is added (for the substrate, quenching by 50 times is observed; for the product, by 6 times). This phenomenon is attributed to the quenching and scattering of light in the presence of the components of blood. It may necessitate the use of blood plasma in order to achieve the highest sensitivity.

To determine the applicability of the method for measuring L-ASNases activity in the complex biological samples, we investigated the influence of various mediums (buffers, blood serum, whole blood, or in liver homogenate samples) and the effect of the type of L-ASNases preparation on the measurement results.

#### 2.1.2. Physicochemical Parameters and Biocatalytic Properties of the Studied L-ASNases Formulations

**Scheme 1:**
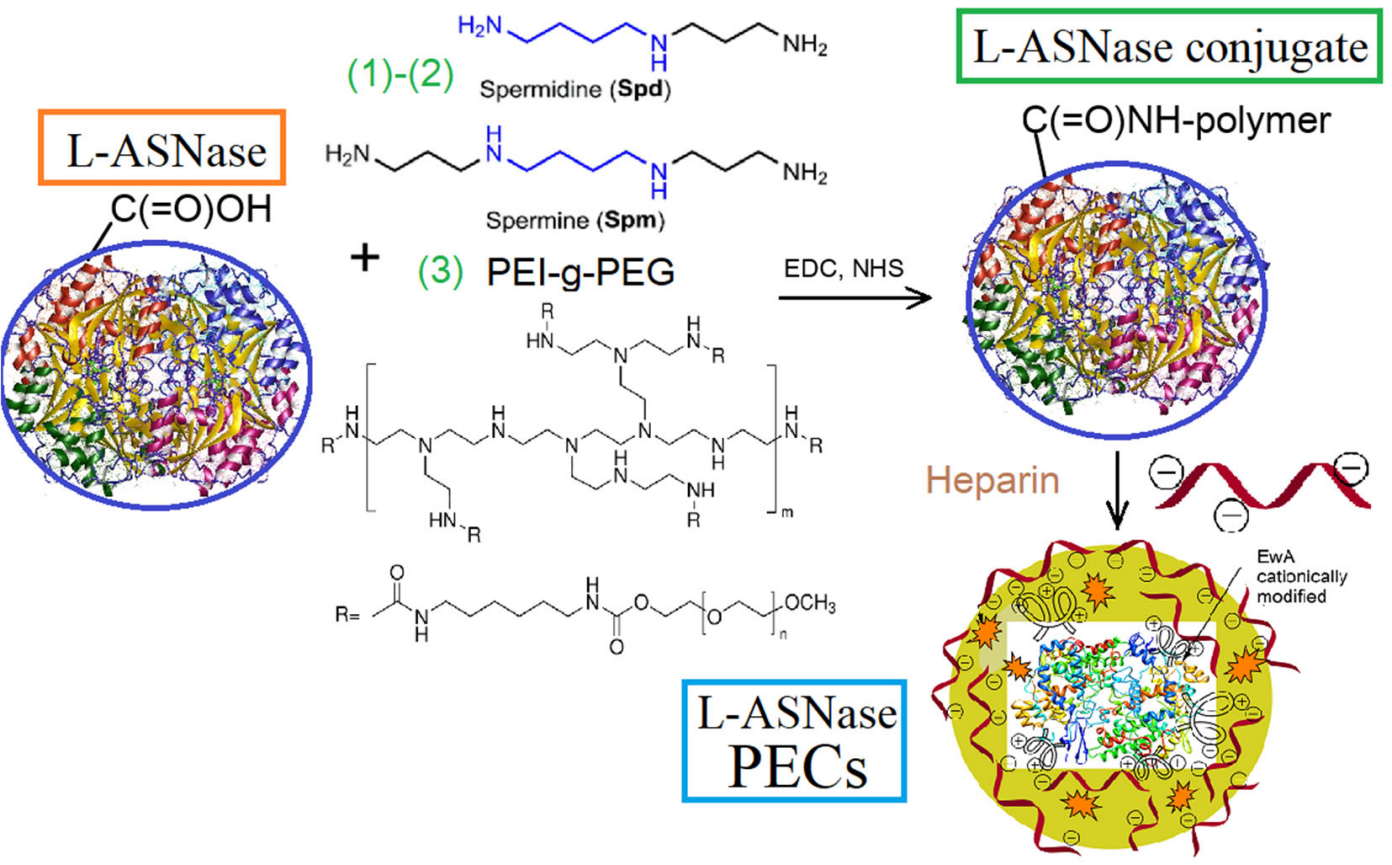
The synthesis schemes of L-ASNase conjugates with cationic polymers and oligoamines, L-ASNase nanogels.

Understanding the behavior of L-ASNase in different formulations is crucial for optimizing its therapeutic efficacy. The formulation can significantly influence the enzyme’s interaction with biological environments, impacting its activity and availability. To investigate this, we prepared various L-ASNase formulations, including native EwA, conjugates of EwA with polycationic polymer, and EwA conjugates in a PEC composition with heparin. The synthesis of these conjugates was achieved through the carbodiimide method, forming an amide bond between the enzyme’s carboxyl groups and the amino groups of the polymers (PEI-g-PEG or spermine), as depicted in Scheme 1.

Successful conjugation was confirmed by FTIR spectroscopy (Supplementary Figure S2). It is crucial to acknowledge that the presence of polymers may potentially interfere with the accuracy of activity measurements, particularly in the context of plasma-based assays, due to their interaction with blood proteins. Consequently, meticulous consideration of these interactions becomes imperative during the analysis process.

The L-ASNases investigated in this study exhibit distinct physicochemical and catalytic properties (Table 1). *Rhodospirillum rubrum* type I L-ASNase (RrA) with a lower molecular weight and a more alkaline pH optimum (pH 9.3) compared to the type II L-ASNases from *E. coli* (EcA) and *Erwinia carotovora* (EwA) (optimal pH 8.0–8.5). EcA and EwA display higher V_max_ values for both Asn or Asp-AMC as substrates, and demonstrate a significantly lower *K*_M_, suggesting higher substrate affinity, than RrA (Supplementary Figure S3).

Modifications of L-asparaginase enzymes, EwA and RrA (Table 2), including spermine conjugation (EwA-sp), PEI-g-PEG conjugation (RrA-PEI-g-PEG), and heparin complexation (PEC), alter their physicochemical properties (zeta potential and molecular weight) and kinetic parameters. Critically, these modifications do not negatively impact substrate affinity, *K*_M_, while significantly enhancing catalytic activity, V_max_. This demonstrates the robustness of this assay for accurately quantifying L-asparaginase activity even in complex formulations and across enzymes with inherently varied *K*_M_ values. This capability is essential for evaluating and optimizing diverse L-asparaginase formulations for therapeutic efficacy.

#### 2.1.3. Optimization of the Conditions for Fluorescence Analysis of L-ASNase Activity

**Figure 2:**
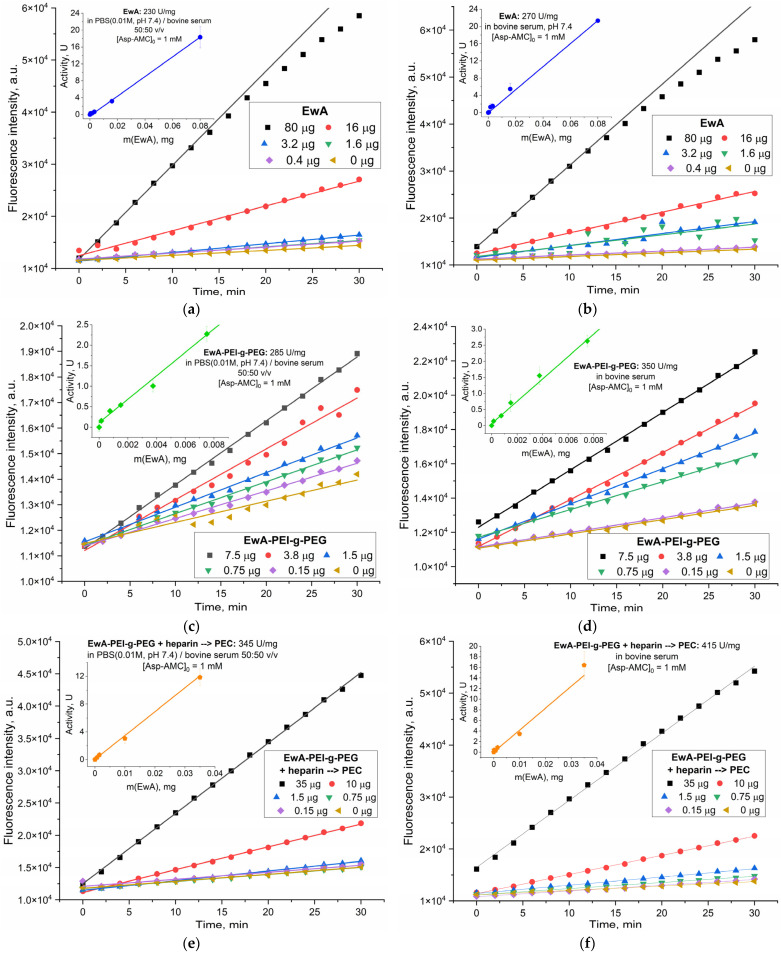
Kinetic curves of Asp-AMC hydrolysis (1 mM) under the action of L-ASNases: (**a**) EwA in 50% (*v*/*v*) bovine serum mixed with PBS; (**a**) EwA in 50% (*v*/*v*) bovine serum mixed with PBS; (**b**) EwA in 100% bovine serum; (**c**) EwA-PEI-g-PEG in 50% (*v*/*v*) bovine serum mixed with PBS; (**d**) EwA-PEI-g-PEG bovine serum; (**e**) EwA-PEI-g-PEG + heparin PEC in 50% (*v*/*v*) bovine serum mixed with PBS; and (**f**) EwA-PEI-g-PEG + heparin PEC in bovine serum. λexci = 360 nm. λemi = 460 nm. pH = 7.4. T = 37 °C. The insert shows the linear dependence of the observed activity on the amount of the enzyme.

Figure 2 presents the kinetic data for L-asparaginase (L-ASNase) activity, using the Asp-AMC hydrolysis assay in both aqueous solution and serum. The observed linear relationships between the rate of Asp-AMC hydrolysis and enzyme concentration demonstrate the assay’s fidelity within the tested range. Background hydrolysis contributes minimally, enabling reliable detection of L-ASNase activity down to tenths of a U/mL. However, enhanced sensitivity, reaching even lower detection limits, could be achieved by extending the incubation period and quantifying accumulated product after 12 or 24 h. Figure 2 demonstrates the successful application of the assay in complex biological matrices like bovine serum, both diluted and undiluted. The linearity of the initial reaction rates (shown in the inserts) confirms the assay’s validity for quantifying enzyme activity.

L-ASNase formulations could significantly affect kinetic properties of the enzyme (Table 2), due to differences in pH optima [19,24], different interactions with plasma proteins, and substrate accessibility. These variations suggest potential differences in the enzyme’s behavior and stability in vitro and in vivo. Native EwA exhibits a Vmax of 280 ± 15 U/mg in PBS Figure 2a). The presence of 50% serum leads to a slight decrease in activity (230 ± 20 U/mg). However, this decrease is not more observed in 100% serum, where the Vmax recovers to 270 ± 10 U/mg. This suggests a sophisticated interplay between serum constituents and the enzyme, including the maintenance of a more active conformation in the context of osmotic pressure exerted by blood proteins and the viscosity of the medium.

Both the EwA-PEI-g-PEG and its heparin PEC formulation show a distinct trend (compared to the native enzyme): the activity increases in the presence of serum. EwA-PEI-g-PEG shows a moderate increase, reaching 350 ± 20 U/mg in 100% serum compared to that in PBS. The PEC formulation with heparin exhibits the most substantial enhancement, with a Vmax of 415 ± 40 U/mg in 100% serum (in PBS 340 ± 25 U/mg). This marked increase suggests that the modifications, particularly the inclusion in the PEC with heparin, could create a more favorable microenvironment for catalysis. This finding has important implications for in vivo applications, where the enzyme will operate in a serum environment.

Table 3 presents the detection limit (LOD, 3σ) and limit of quantification (LOQ, 10σ) for various L-ASNase formulations in different media. The LOD is the lowest concentration of ASP that can be reliably distinguished from background noise, typically calculated as three times the standard deviation of the blank. The LOQ, representing the lowest concentration that can be quantified with acceptable accuracy and precision, is typically calculated as 10 times the standard deviation of the blank.

In L-ASNase therapy, serum activity typically peaks around 5 U/mL immediately after administration and declines to below 100 U/L within 5–25 days, depending on the formulation [36]. So, the methodology should allow us to determine the activity unit, at least 100 U/L, to detect a therapeutically significant amount of Asparaginase activity in the plasma.

The data in Table 3 clearly demonstrate a significant improvement in both LOD and LOQ when the assay is performed in 100% serum. In PBS, the LOD and LOQ are 0.17 U/mL and 0.57 U/mL, respectively, irrespective of the L-ASNase formulation. This translates to a detectable limit of approximately 0.5–1 μg/mL of enzyme, enabling pharmacokinetic studies capable of quantifying down to 10% of the administered activity.

When assessing L-ASNase activity based on the initial interval of the kinetic curve, it allows for accurate quantification of the target enzyme’s activity, achieving a limit of detection (LOD) in serum blood of 40 U/L and a limit of quantification (LOQ) of 130 U/L. So, the detection limit is lower in plasma than in PBS buffer. This difference is due to the fact that the fluorescence intensity of both Asp-AMC and AMC exhibits a significant variation depending on the reaction medium (Figure 1a). In blood serum, the fluorescence intensity of substrate Asp-AMC is found to be 40% lower compared to that in PBS. Conversely, for AMC (reaction product), the maximum intensity is 15% higher in serum than in PBS. Thus, we obtain a higher product/substrate ratio (>10:1) for blood serum compared to that in PBS, which reduces LOD, and LOQ.

Conversely, employing a product accumulation strategy over an extended period, such as 12 h, substantially enhances the assay’s sensitivity, leading to significantly lower LOD and LOQ values across all tested conditions. The most pronounced improvement is observed in PBS buffer, where an LOD of 10 U/L and LOQ of 33 U/L are achieved. This superior performance in buffer is principally attributed to the absence of endogenous serum enzymes (e.g., other proteases or amidases) that could contribute to non-specific asparagine or Asp-AMC hydrolysis over longer incubation times. In serum, these interfering activities become more significant with prolonged incubation, leading to higher background signals and thus slightly elevated LODs (21–26 U/L) and LOQs (70–86 U/L) for the 12-h accumulation method compared to PBS, yet these are still markedly better than the initial rate measurements in serum

Crucially, our fluorescence assay achieves an LOQ of 26 U/L (0.026 U/mL) in 100% serum. This enhanced sensitivity in a biologically relevant matrix offers a significant advantage for monitoring asparaginase levels in patients for up to 2–3 weeks post-treatment. In contrast, the Nessler assay, while possessing a reported sensitivity of ~100 U/L in buffer, is unsuitable for serum due to its non-specific reactivity with various serum components (amines, hydroxyls, etc.) [37]. Therefore, the fluorescence assay provides a critical tool for accurate and sensitive monitoring of therapeutic asparaginase activity throughout the clinically relevant timeframe.

Previously, we developed a method based on the circular dichroism (CD) spectroscopy for studying the activity of native and conjugated enzymes [20,21]. Here, we applied CD spectroscopy as a reference method to verify the activity determined by a novel fluorescence method. The results obtained are in agreement with that obtained by CD spectroscopy for the reference native EwA enzyme in aqueous solution.

#### 2.1.4. Calibration of L-ASNase Activity in Various Buffer/Serum System

Optimizing assay conditions for accurate measurement of L-asparaginase activity in serum is paramount. Our analysis revealed a nonlinear relationship between enzyme activity, enzyme concentration, and serum composition, as visualized in the 3D activity diagrams. The output of three-dimensional visual representations of L-ASNase activity is contingent upon a variety of factors, including enzyme concentration and the composition of serum.

**Figure 3:**
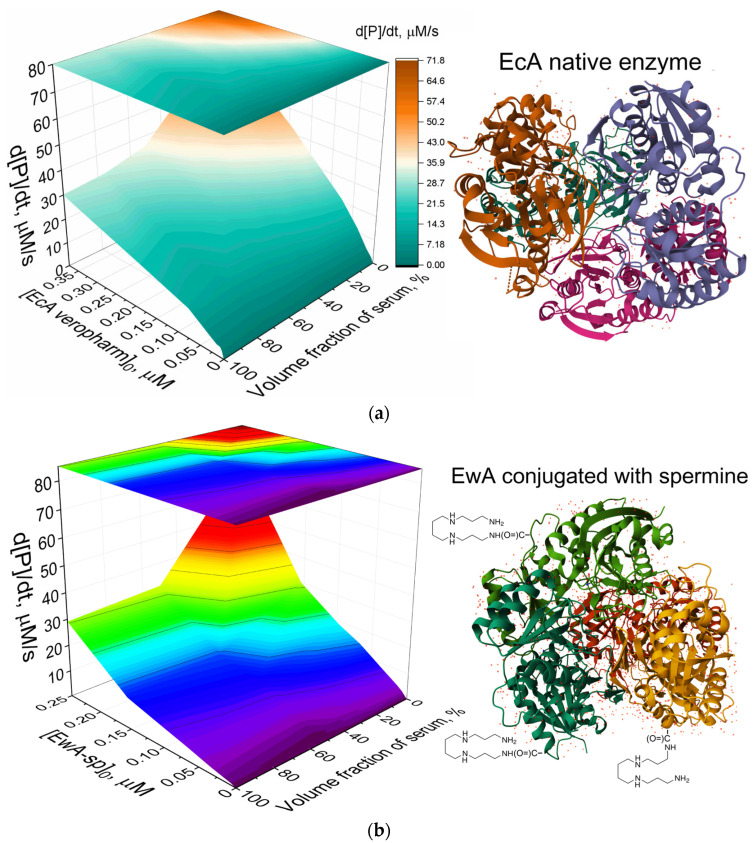

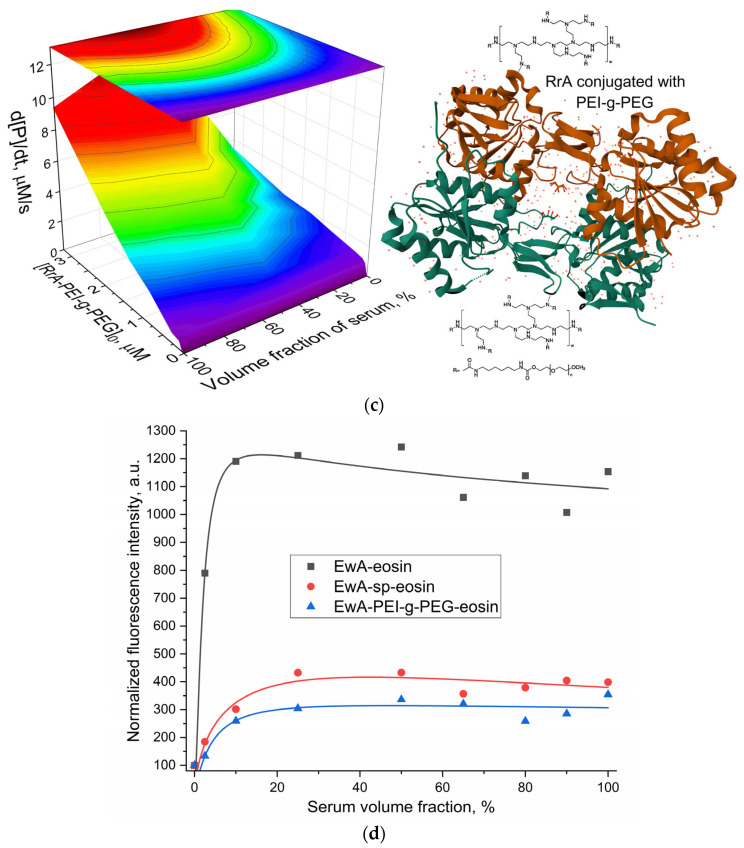
Three-dimensional graphs of the relationship between the rate of enzymatic hydrolysis of Asp-AMC by (**a**) the native EcA enzyme, (**b**) EwA conjugated with spermine, and (**c**) RrA conjugated with PEI-g-PEG for determining the optimal composition of the analyzed buffer/serum system. C_0_(Asp-AMC) = 1 mM. λexci = 360 nm. λemi = 460 nm. pH = 7.4. T = 37 °C. (**d**) Dependence of the fluorescence intensity of eosin covalently attached to asparaginase for the formation of native EwA, EwA-sp conjugate, and EwA-PEI-g-PEG conjugate on the volume fraction of blood serum. C(L-ASNase) = 0.1 mg/mL. λexci = 515 nm. λemi = 550 nm. pH = 7.4. T = 37 °C.

Figure 3 presents three-dimensional graphs illustrating the relationship between the enzymatic hydrolysis rate, enzyme concentration, and serum volume fraction for Asp-AMC hydrolysis catalyzed by (a) native EcA, used here as a commercially available control; (b) spermine-conjugated EwA (EwA-sp); and (c) PEI-g-PEG-conjugated RrA. To investigate the influence of enzyme properties, such as molecular mass (MM) and isoelectric point (pI), on serum interactions and assay performance, we expanded our analysis to include additional L-asparaginases. Specifically, we chose enzymes with contrasting characteristics: RrA (tetrameric Mw of approximately 18 × 4 = 72 kDa and pI of 5.0–5.2) and EwA (tetrameric Mw of approximately 34 × 4 = 136 kDa and pI of 7.5–8.0). This comparison allows us to assess how these properties affect the 3D diagram of serum influence (Table 1 and Table 2) and, ultimately, the accuracy of activity measurements in a complex biological matrix. These graphs were generated to determine the optimal composition of a buffer/serum system for analysis. Native EcA serves as a reference sample due to its widespread use and established activity profile. This approach enables optimization of the assay conditions for each enzyme variant, ensuring accurate and reliable activity measurements in a biologically relevant context.

Native EcA enzyme

The data presented in Figure 3a elucidate the impact of enzyme concentration and serum volume fraction on the enzymatic hydrolysis rate of Asp-AMC by native EcA. In a serum-free buffer, the reaction rate increases proportionally with enzyme concentration, demonstrating a classic enzyme kinetics profile. For instance, increasing the EcA concentration from 0.038 µM to 0.075 µM results in an approximately two-fold increase in the hydrolysis rate (from 11.8 µM/s to 21.2 µM/s). The introduction of serum into the buffer system exerts a significant effect on EcA activity: the presence of serum leads to a marked decrease in the reaction rate. This effect is particularly pronounced at higher enzyme concentrations. For example, at 0.38 µM EcA, the hydrolysis rate in the presence of 35% serum (45 µM/s) is more than two-fold lower compared to the serum-free rate (72 µM/s). Even at lower enzyme concentrations, such as 3.8 nM, the presence of 75% serum reduces the hydrolysis rate to 1.25 µM/s, compared to 1.86 µM/s in the absence of serum. The observed effect of serum suggests active interactions between serum components and EcA, leading to reduced catalytic efficiency.

EwA conjugated with spermine

The data presented on Figure 3b elucidate the impact of enzyme concentration and serum volume fraction on the enzymatic hydrolysis rate of Asp-AMC by EwA conjugated with spermine (EwA-sp). Both EwA and EcA, type II periplasmic L-asparaginases, exhibit similar activity profiles regarding Asp-AMC hydrolysis, as evidenced by the three-dimensional activity landscapes. For both enzymes, increasing enzyme concentration correlates with a higher hydrolysis rate in a serum-free environment. EwA-sp demonstrates a linear response to increasing concentrations, with a rate increase from 2.3 µM/s at 0.007 µM to 72 µM/s at 0.25 µM. Similar to native EcA, the presence of serum significantly effects the EwA-sp activity. For example, at 0.25 µM EwA-sp, 50% serum reduces the hydrolysis rate to 31 µM/s, representing a greater than two-fold decrease compared to the serum-free rate. Nonetheless, as we have previously discussed, the EwA conjugate with a high molecular weight of PEI-g-PEG does not only fail to reduce, but rather significantly enhances activity in 50% of the serum. This polymer actively enhances the properties of the enzyme within the serum. Conversely, the modification of EwA with spermine (EwA-sp) appears to minimally impact the 3D activity profile, maintaining the characteristic serum inhibition pattern observed with native EcA and EwA (periplasmic enzymes). The data suggest that serum components likely interact with both enzymes in a comparable manner, leading to diminished catalytic efficiency.

RrA conjugated with PEI-g-PEG

The data presented on Figure 3c elucidate the impact of enzyme concentration and serum volume fraction on the enzymatic hydrolysis rate of Asp-AMC by RrA conjugated with PEI-g-PEG. In contrast to the periplasmic type II L-asparaginases, EcA and EwA, the cytosolic type I L-asparaginase RrA in native form (Table 2), and when it is conjugated with the cationic copolymer PEI-g-PEG (RrA-PEI-g-PEG), exhibits a drastically different activity profile (Figure 3c). While EcA and EwA-sp demonstrate reduced activity in serum, RrA-PEI-g-PEG (the same effect is observed for EwA-PEI-g-PEG) shows enhanced activity in the presence of serum, with an optimal serum volume fraction between 60 and 100%. The large cationic polymer could alter surface charge interactions with serum albumin, masking epitopes that might otherwise inhibit activity or even promoting favorable interactions that enhance substrate binding or stabilize active RrA enzyme conformations. For instance, at 3.3 µM RrA-PEI-g-PEG, the hydrolysis rate increases from 3.40 µM/s in serum-free buffer to 12.1 µM/s in 50% serum and 9.3 µM/s in 100% serum (a similar situation is observed for the native RrA enzyme, but to a lesser extent—Table 2). This suggests that the PEI-g-PEG modification may mediate favorable interactions with serum components, potentially enhancing substrate binding or enzyme conformation, leading to increased activity. This distinct activity profile highlights the potential of polymer conjugation to modulate enzyme behavior and overcome serum inhibition, offering promising avenues for improving L-asparaginase therapy.

Interactions of L-ASNases with serum proteins

The fluorescence technique is sensitive to changes in proximity and environmental conditions, making it relevant for studying biomolecular interactions such as protein–ligand binding and protein–protein association [38,39,40,41].

In our research, we employ fluorescence signal changes using a covalently attached eosin label on L-asparaginase to investigate interactions with blood proteins. Eosin’s fluorescence is particularly responsive to its microenvironment, protein binding [42]. Eosin’s absorption maximum shifts from 514 nm to 530 ± 5 nm upon protein binding, with this bathochromic shift being proportional to protein concentration. This interaction occurs via electrostatic binding between the dye’s carboxylic/phenolic groups and specific amino acid residues, like arginine, histidine, lysine, and tryptophan. The resulting stable water-soluble protein–dye complex enables quantitative protein estimation, as binding constants vary with protein type and solution pH [42]. By observing changes in eosin fluorescence, we can detect and quantify the interactions between modified asparaginase and components of the blood proteins.

To elucidate the impact of L-ASNase conjugation on its activity in serum, the dependencies of the fluorescence intensity of covalently bound eosin with L-ASNase were plotted for different formulations, native EwA, EwA-sp conjugate, and EwA-PEI-g-PEG conjugate, on the volume fraction of serum—Figure 3d.

An increase in the volume fraction of serum leads to an ignition of eosin fluorescence, indicating an interaction between serum proteins and L-ASNase. Native L-ASNase exhibits a 13-fold increase, suggesting a significant interaction with serum components. Conjugation of L-ASNase with polymers, on the other hand, reduces the degree of interaction with serum: spermine and PEI-g-PEG conjugates show lower fluorescence ignition, 4-fold and 3-fold, respectively, compared to the native enzyme (12-fold), suggesting the shielding effect of the protein surface from the plasma proteins. This shielding appears to limit the interaction between the serum proteins and eosin molecules in the conjugates.

Our investigation reveals that conjugation of L-asparaginase, especially with cationic polymers such as PEI-g-PEG, effectively shields the enzyme surface (with eosin as a marker) from the interaction with serum proteins (Figure 3d). Polymer protective effect mitigates the typical activity loss observed with native L-asparaginase in blood serum. This observation aligns with the existing literature demonstrating that in PEGylated liposomes, bovine serum albumin (BSA) predominantly interacts with the PEG chains rather than the liposomal surface itself [43]. This distinction is crucial, as it suggests a similar mechanism in our system. We hypothesize that the interaction between BSA and the PEI-g-PEG conjugation potentially enhances L-asparaginase activity, while direct BSA interaction with the native enzyme leads to activity reduction. This can be attributed to the negative zeta potential of BSA (approximately −12 mV), which can impart a negative charge to the enzyme, potentially disrupting its quaternary structure and thus its activity [26]. The PEI-g-PEG coating, however, appears to prevent this charge transfer, preserving the structural integrity and activity of L-asparaginase. Potentially this effect could lead to improved stability and extended circulation time in vivo.

#### 2.1.5. Validation of the Developed Methodology Using Test Samples According to the “Entered–Found” System

**Figure 4:**
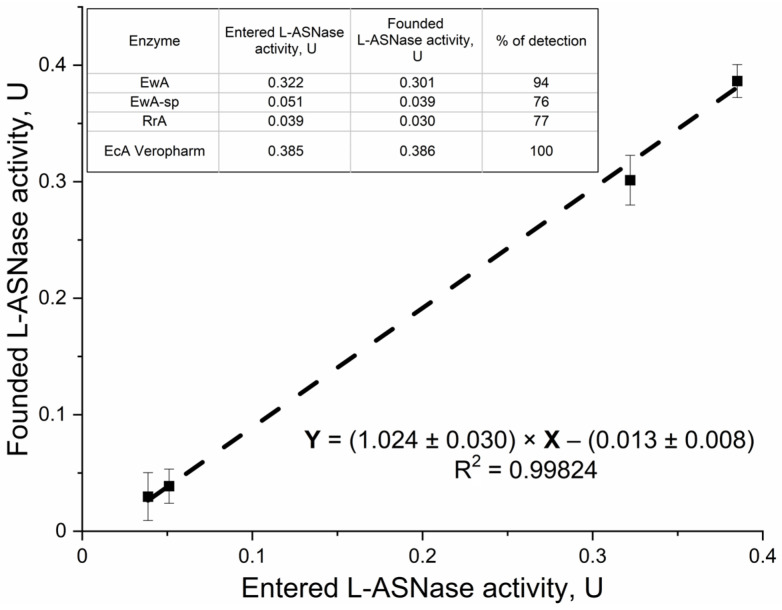
Validation (verification) of the developed methodology using L-ASNase (EwA, EwA-sp, RrA, and EcA Veropharm) test samples. C_0_(Asp-AMC) = 1 mM. λexci = 360 nm. λemi = 460 nm. Bovine serum. pH = 7.4. T = 37 °C. The points on the graph are experimental samples (3 replicates, ±SD). The dotted line is the trend line.

Figure 4 presents a validation of the L-ASNase activity assay, using various L-ASNase samples (EwA, EwA-sp, RrA, and EcA Veropharm). This diverse set of samples allowed us to assess the influence of the source organism and polymer conjugation on the assay’s performance. Our objective was to evaluate the assay’s accuracy and reliability across different enzyme formulations and dilutions, particularly in the presence of serum, given its potential impact on activity measurements. Using the calibration established in Figure 1 (for both buffer and serum), we determined the activities of these samples in varying serum concentrations and compared them to their expected activities (based on mg of enzyme) determined by CD spectroscopy. The data demonstrate good agreement between the entered (expected) and founded (measured) enzyme activities, supporting the reliability and accuracy of the developed methodology. Specifically, the percentage of detection, reflecting the ratio of founded to entered activity, ranges from 75.9% to 100.4%, indicating efficient detection of the enzyme activity across different L-ASNase types. The EcA Veropharm sample displays complete recovery (100.4%), suggesting optimal assay performance for this particular enzyme. While demonstrating good overall recovery, the EwA-sp and RrA samples exhibit slightly lower detection percentages (75.9% and 77.1%, respectively)—because when low concentrations of asparaginase drugs are administered, a reduced % of the determination is observed.

The close agreement between the input and measured activities, reflected in the strong linear correlation entered vs. found) (Y = (1.024 ± 0.030) × X − (0.013 ± 0.008), R^2^ = 0.99824), confirms the assay’s accuracy and reliability. The near-unity slope (1.024 ± 0.030) indicates minimal systematic error and close to 100% of the activity found, while the small y-intercept (−0.013 ± 0.008) suggests negligible background signal or interference. This observation is crucial for interpreting pharmacokinetic data, as measurements in serum may overestimate the true in vivo activity. This rigorous validation, coupled with the low detection limit (around 0.03 U), establishes the fluorometric assay as a robust and sensitive tool for quantifying L-ASNase activity, critical for applications ranging from therapeutic dose optimization to the characterization of novel L-ASNase formulations.

### 2.2. In Situ Monitoring of L-ASNase Activity in Complex Biological Matrices Using FTIR Spectroscopy

#### 2.2.1. The Salient Characteristics of Employing FTIR Spectroscopy for the Assessment of L-ASNase Activity

While fluorescence-based assays using substrates like Asp-AMC have been widely employed for high-throughput screening of L-asparaginase activity, they offer limited insights into the detailed enzymatic mechanisms and are often incompatible with complex biological matrices like whole blood. In this section, we present an approach using FTIR spectroscopy to study L-asparaginase activity in detail. Crucially, FTIR spectroscopy offers compatibility with diluted whole blood samples, overcoming the limitations of fluorescence assays and providing a more physiologically relevant environment for studying enzyme activity, potentially bridging the gap between in vitro screening and in vivo properties.

The L-ASNase activity measurements using FTIR spectroscopy provide valuable insights into the enzyme’s catalytic mechanism and the influence of the biological environment. The FTIR spectroscopy method makes it possible to detect the Asn hydrolysis process by changing the characteristic absorption bands associated with the hydrolysis of the amide bond. In the case of Asn and Asp, this is the band of amide I (1680 cm^−1^) in the spectrum of Asn, corresponding to the absorption of the amide bond, and the band at 1590 cm^−1^, which corresponds to the absorption of carboxyl groups of Asp (Supplementary Figure S4).

**Figure 5:**
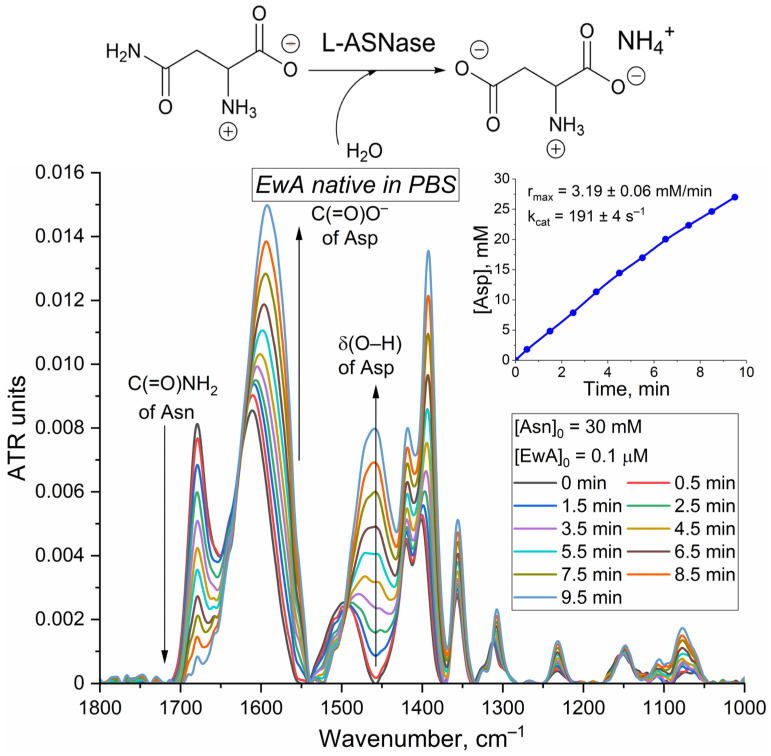
FTIR spectra of asparagine (30 mM) during catalytic hydrolysis by EwA (100 nM). The insert shows the kinetic curve of hydrolysis. Sodium-phosphate buffer (0.1 M, pH 7.4). T = 37 °C.

**Figure 6:**
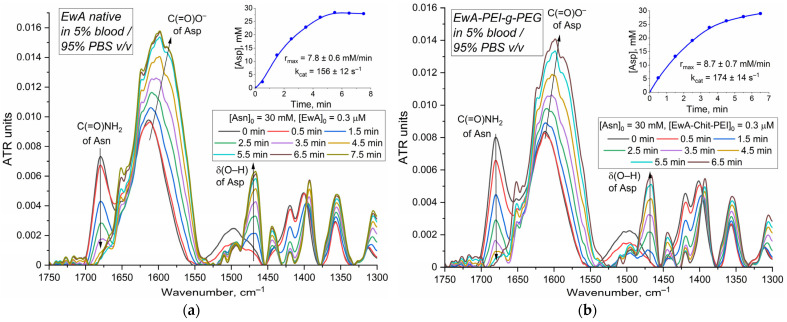

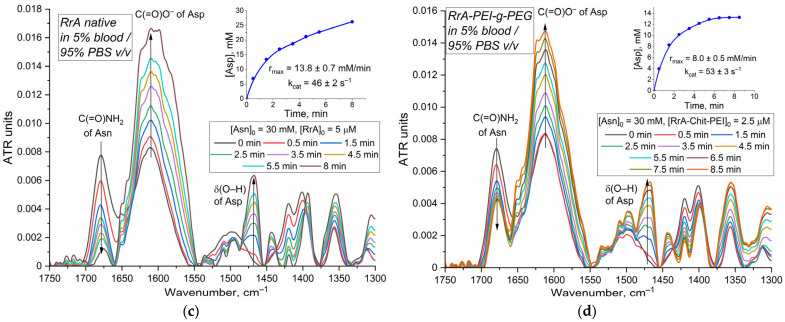
FTIR spectra of asparagine (30 mM) during catalytic hydrolysis by (**a**) EwA (300 nM) and (**b**) EwA conjugated with PEI-PEG (300 nM) in 5% blood (+95% PBS *v*/*v*). The insert shows the kinetic curve of hydrolysis. FTIR spectra of asparagine (30 mM) during catalytic hydrolysis by (**c**) RrA (5 µM) and (**d**) RrA conjugated with PEI-PEG (2.5 µM) in 5% blood (+95% PBS *v*/*v*). The insert shows the kinetic curve of hydrolysis. The contribution of blood cells is taken into account as the background (Supplementary Figure S5). PBS (0.01 M, pH 7.4). T = 37 °C.

Figure 5 presents the FTIR spectra of Asn at 30 mM concentration during the process of catalytic hydrolysis by EwA. The observed spectral changes during asparagine hydrolysis (decrease at 1680 cm^−1^ and increases at 1460 cm^−1^, 1590 cm^−1^, and 1400 cm^−1^) reflect the conversion of Asn to Asp. The 1680 cm^−1^ band corresponds to the amide C=O stretch in Asn, which diminishes as the amide bond is hydrolyzed. The increases at 1460 cm^−1^, 1590 cm^−1^, and 1400 cm^−1^ represent emerging carboxylate (COO⁻) oscillations in the aspartate product. To construct kinetic curves, we used a band 1680 cm^−1^ corresponding to C(=O)NH_2_ of Asn (Supplementary Figure S4). By tracking the decrease in this band’s intensity, we converted the calibration curves ATR units into concentration units. Then, we constructed the Asp product accumulation (Figure 5 insert) curves. By analyzing the tangent of the angle of inclination in the initial section, we determined Vmax and subsequently calculated k_cat_ (Figure 6a,b).

These specific spectral changes serve as markers for monitoring the reaction progress and determining enzyme activity.

FTIR spectroscopy, by monitoring changes in characteristic absorption bands associated with amide bond hydrolysis, provides a direct measure of L-asparagine conversion to L-aspartate. The calculated *k*_cat_ (191 ± 4 s^−1^) and V_max_ (337 ± 7 U/mg) for native EwA using FTIR align well with previously published data obtained through CD spectroscopy [44].

In the case of fluorimetry, employing the substrate analog Asp-AMC yields a slightly lower Vmax value of 280 ± 15 U/mg for EwA in PBS. This discrepancy is expected and can be attributed to the difference in substrates. Asp-AMC, while useful for its fluorescent properties, is not identical to the natural substrate, L-asparagine, and the enzyme may exhibit different kinetic parameters when interacting with it. The fluorimetry measurement is in the same order of magnitude as the FTIR, and CD spectroscopy data confirm its suitability as a complementary method, especially for situations where continuous monitoring or higher throughput is needed. Therefore, using a combination of these methods, each with its own strengths and limitations, allows for a comprehensive and validated assessment of L-ASNase activity, enabling confident progression to further studies.

#### 2.2.2. Catalytic Activity of L-Asparaginases in Whole Blood Systems

Different L-asparaginases exhibit varying kinetic properties, substrate affinities (*K*_M_), and oligomeric states, all of which can influence their behavior in different assay conditions and affect the sensitivity of the chosen detection method. Therefore, it is crucial to assess the applicability and performance of a given method across a range of L-ASNases. This includes evaluating the method’s ability to accurately determine *K*_M_ values and how different assay conditions might impact these measurements. For instance, while fluorescence-based assays offer sensitivity for measuring L-ASNase activity, they are susceptible to quenching in complex biological matrices like whole blood. This necessitates exploring alternative methods such as FTIR spectroscopy, which can operate effectively in optically opaque environments. By examining L-ASNase activity in whole blood using FTIR, we can evaluate the influence of the biological milieu on both native and modified enzymes, as demonstrated in Figure 6.

Figure 6a,b illustrate L-asparagine hydrolysis by native and EwA-PEI-g-PEG, respectively. The kinetic inserts reveal comparable hydrolysis rates for both forms, corroborating the findings from the three-dimensional activity profiles (Figure 4) and suggesting a moderate (~10%) improvement in activity in blood upon PEI-PEG modification.

Figure 6c,d show asparagine hydrolysis by native RrA and RrA-PEI-g-PEG. Interestingly, the conjugated RrA exhibits a higher hydrolysis rate than the native enzyme, potentially indicating a beneficial effect of modification on activity within the blood environment.

The data shown in Figure 6 (inserts) present a comparison of the catalytic constants (k_cat_) for different L-asparaginases (EwA, both native and modified with PEI-g-PEG) and RrA (native and modified) in different environments (PBS and 5% blood/95% PBS). Native EwA exhibits a higher k_cat_ in PBS (191 s^−1^) compared to the 5% blood/95% PBS mixture (156 s^−1^), (Vmax decrease from 337 U/mg to 275 U/mg). This decrease in *k*_cat_ suggests the interference by components within the blood. The PEI-g-PEG modification of EwA further increases the *k*_cat_ in the blood-containing mixture (174 s^−1^) and increases the V_max_ value to 307 U/mg, indicating that the modification protects the enzyme from the effect of serum protein. A similar trend was observed using fluorometric assays. Our investigation reveals that conjugation of L-asparaginase, especially with cationic polymers such as PEI-g-PEG, effectively shields the enzyme from interaction with serum proteins (Figure 3d). Polymer protective effect mitigates the typical activity loss observed with native L-asparaginase in blood serum. This observation aligns with the existing literature demonstrating that in PEGylated liposomes, bovine serum albumin (BSA) predominantly interacts with the PEG chains rather than the liposomal surface itself [43]. This distinction is crucial, as it suggests a parallel mechanism in our system. We hypothesize that the interaction between BSA and the PEI-g-PEG conjugate potentially enhances L-asparaginase activity, while direct BSA interaction with the native enzyme likely leads to activity reduction. This can be attributed to the negative zeta potential of BSA (approximately −12 mV), which can impart a negative charge to the enzyme, potentially disrupting its quaternary structure and thus its activity. The PEI-g-PEG coating, however, appears to prevent this charge transfer, preserving the structural integrity and activity of L-asparaginase. This shielding effect offered by the PEI-g-PEG polymer represents a promising strategy for enhancing the stability and efficacy of L-asparaginase in therapeutic applications.

The kinetic parameters measured in blood and serum are generally consistent with previously reported values in aqueous buffer systems; for example, the V_max_ values for native EwA (490 ± 20 U/mg) and RrA (46 ± 3 U/mg) align well with the literature values [24]. This agreement validates the accuracy of our assay and emphasizes that while blood components can influence activity, the assay reliably quantifies these changes, providing crucial information for optimizing therapeutic formulations.

The capacity of FTIR spectroscopy to determine L-ASNase activity in diluted whole blood (up to 20% *v*/*v*, beyond which the background contribution from erythrocytes becomes excessive) offers a significant advantage, enabling measurements in a more physiologically relevant environment. The study’s findings underscore several key aspects regarding L-ASNase activity and the influence of both biological environment and enzyme modification. The presence of blood components can impact L-ASNase activity, as evidenced by the slightly reduced *k*_cat_ and V_max_ values observed for native EwA and RrA in blood compared to PBS. Modification with polycations (like PEI-g-PEG) appears to mitigate the effects of blood components, leading to increased *k*_cat_ values for RrA in blood.

The use of FTIR spectroscopy provides a valuable tool for evaluating enzyme performance in complex biological matrices and contributes to a more comprehensive understanding of L-ASNase behavior in vivo.

### 2.3. The Application of the Developed Methodology for In Vivo Pharmacokinetic Analysis: A Study of L-Asparaginase Activity in Murine Models

The validation of methods for measuring asparaginase activity relies heavily on research conducted in murine models. Here, the in vivo activity of L-asparaginase is measured in in vivo pharmacokinetic studies using mouse models. We examined residual L-ASNase activity in mouse serum 20 days after a 10-day course of intraperitoneal administration of either native EwA or the EwA–spermine conjugate (EwA-sp) at a dosage of 4000 U/kg intraperitoneally daily (6 mice per group). This time point was chosen to assess long-term persistence and potential extended therapeutic benefits. Enzyme activity was quantified fluorometrically, using the Asp-AMC substrate, allowing for direct measurement in undiluted serum.

The data show that after 10 days of daily dosing (4000 U/kg), the EwA-sp conjugate accumulates to significantly higher serum activity levels (10 ± 1 U/mL) compared to native EwA (4.9 ± 0.4 U/mL). This observation strongly suggests that the chemical modification of EwA results in improved pharmacokinetic properties, likely involving a prolonged circulation half-life in the bloodstream, leading to greater accumulation over the treatment period.

Table 4 also provides measurements of “residual activity” 20 days after the 10-day treatment. It is notable that significant background asparaginase activity is detected in the serum of control mice at this late time point (average 0.56 U/mL), indicating the presence of endogenous activity or assay interference. Subtracting this background activity (as shown in the “minus background” section) reveals that the residual activity attributable to the administered enzyme forms is very low 20 days post-treatment. While the average “minus background” value for EwA-sp (0.08 ± 0.08 U/mL) is slightly higher than that for native EwA (0.03 ± 0.02 U/mL), the high standard deviation for EwA-sp suggests considerable variability among individual mice at this very low level of detection above background.

A key challenge in pharmacokinetic studies of L-asparaginase is detecting residual activity, especially given the limitations of standard assays with detection limits of 0.1–0.5 U/mL. Our fluorescence-based method, employing Asp-AMC and leveraging overnight incubation to accumulate the fluorescent product, achieves significantly enhanced sensitivity, with detection limits as low as 0.01–0.05 U/mL. This improved sensitivity is crucial for tracking trace amounts of active enzyme, particularly in the context of long-term pharmacokinetic studies. Given the reported half-lives of L-asparaginases (ranging from hours for native enzymes to days for PEGylated forms), this enhanced sensitivity allows for more accurate assessment of long-term activity, which is essential for optimizing dosing regimens, minimizing overall drug exposure, and potentially reducing immunogenicity. We anticipate that our novel formulations will exhibit prolonged activity, further emphasizing the need for highly sensitive detection methods like the one presented here.

**Figure 7:**
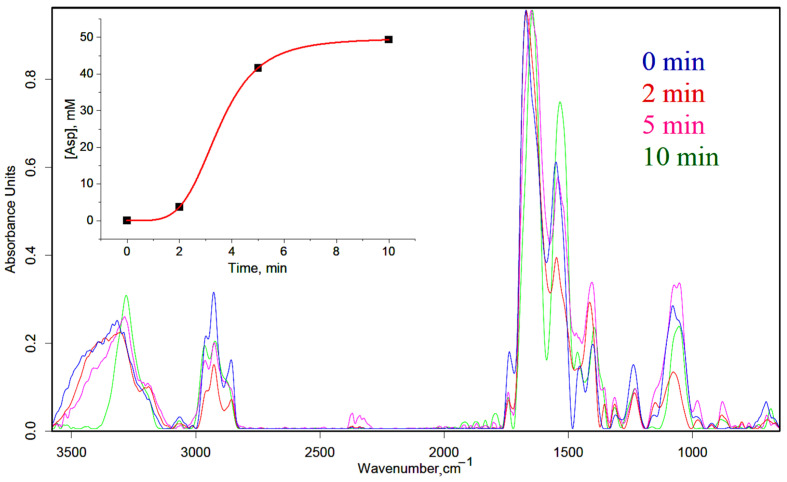
The experiment “introduced and found” in rat liver homogenate. In total, 0.012 mg of the enzyme was introduced to rat liver homogenate (450 µL liver homogenate suspension of 100 mg tissue). Normalized FTIR spectra of asparagine hydrolysis with an aliquot of 2 µL EwA in 40 µL substrate solution. pH 7.4. T = 37 °C.

Understanding enzyme activity within specific organs is crucial for pharmacological studies. Figure 7 showcases the application of FTIR spectroscopy to monitor L-asparaginase activity in a complex biological matrix, specifically rat liver homogenate, using an “introduced and found” experimental design. The figure displays normalized FTIR spectra acquired during the hydrolysis of asparagine by an aliquot of liver homogenate (derived from 100 mg of tissue with added EwA). The key spectral change tracked is the shift of the asparagine v(C=O) peak from 1670 cm^−1^ to 1645 cm^−1^, indicative of asparagine hydrolysis. A known quantity of enzyme (0.012 mg/mL of EwA, corresponding to 3.4 U/mL in PBS and 3.2 U/mL in serum based on the calibrations) was introduced into the homogenate. The measured activity in the liver homogenate was 2.2 U/mL, suggesting partial inactivation of EwA within this complex biological environment. This observed decrease in activity highlights the potential challenges of directly extrapolating in vitro activity measurements (e.g., in PBS or serum) to in vivo situations. The ability to directly measure enzyme activity within tissue homogenates, as demonstrated here, offers valuable insights into the enzyme’s behavior in a more realistic biological context.

It should be noted that upon the catalytic reaction of asparagine hydrolysis, we observe a distinct and significant decrease in the characteristic Asn peak located around 1680 cm^−1^, accompanied by intensifying peaks characteristic of the aspartate carboxylate group (COO^−^) at 1580 cm^−1^, 1460 cm^−1^, and 1380 cm^−1^ (Supplementary Figure S6a). In the absence of the enzyme, we do not observe a change in the intensity of these analytically significant peaks (Supplementary Figure S6b).

Validating ex vivo methods, like the one employed here, is crucial for ensuring the accuracy and reliability of activity measurements in complex biological samples. Demonstrating that the observed activity differences are indeed due to the inherent properties of the formulations and not artifacts of the measurement technique is paramount. In this study, the successful application of the fluorimetry assay to detect residual L-ASNase activity in serum provides valuable insights into the in vivo behavior of these enzymes. Finally, correlating these residual activity measurements with therapeutic outcomes would be crucial to determine the clinical relevance of these findings. The extended activity observed with EwA-sp holds particular promise for improving treatment strategies in pediatric acute lymphoblastic leukemia, where minimizing the frequency of injections and reducing the associated side effects are of paramount importance.

Table 5 compares methods for determining L-asparaginase activity. While most of the methods can measure specific activity, their ability to analyze samples in complex biological media (like blood serum) and evaluate enzyme conjugates varies significantly.

The table highlights that fluorimetry with Asp-AMC and FTIR spectroscopy are uniquely suited for measuring L-asparaginase activity directly in biological samples and for analyzing modified enzyme formulations. In contrast, methods like Nesslerization, pH-station, and conductometry are shown to be unsuitable for such complex matrices. This comparison underscores the practical advantages of fluorimetry with Asp-AMC, particularly for pharmacokinetic studies and the characterization of novel enzyme preparations in biological contexts, due to its capability to handle complex samples where other methods are failed.

## 3. Materials and Methods

### 3.1. Chemicals

L-ASNase fluorescent substrate L-aspartic acid β-(7-amido-4-methylcoumarin) (Asp-AMC) and product 7-amino-4-methylcoumarin AMC were purchased from Sigma-Aldrich (St. Louis, MO, USA). Polymers for enzyme modification, PEI (2 kDa), PEI-g-PEG (PEI 25 kDa, PEG 5 kDa), and oligoamine spermine (sp), were purchased from Sigma-Aldrich (St. Louis, MO, USA). Heparin (12–14 kDa) was used medically for intravenous administration.

Other compounds, 1-ethyl-3-(3-dimethylaminopropyl) carbodiimide (EDC), N-hydroxysuccinimide (NHS), salts, acids, and buffer components, were from Reachim (Moscow, Russia).

### 3.2. L-Asparaginases

*E. coli* L-ASNase (EcA) was purchased from Veropharm^®^. Recombinant L-ASNase from *Erwinia carotovora* (EwA) was obtained as described earlier [50]. The *Rhodospirillum rubrum* L-ASNase (RrA) enzyme was obtained as described earlier [22]. The initial activity of L-ASNases was determined using the standard method of circular dichroism spectroscopy on device J-815 CD spectrometer (Jasco, Tokyo, Japan) [20,22].

### 3.3. Synthesis and Characterization of L-ASNase Conjugates and PECs

L-ASNase conjugates were prepared via carbodiimide chemistry. Briefly, L-ASNase (1 mg/mL) in 0.02 M phosphate buffer (pH 6.0) was activated with EDC and NHS (dissolved in acetonitrile at 30 mg/mL) at a weight ratio of 0.4 and 0.2, respectively, relative to the enzyme. Following a 30-min incubation at 35 °C, a solution of either PEI-g-PEG, or spermine in the same buffer was added dropwise to the activated L-ASNase until a 2:1 enzyme-to-polymer mass ratio was achieved. The reaction mixture was then incubated for an additional 2 h at 35 °C. The resulting conjugates were purified by dialysis (12–14 kDa cutoff) against PBS at 4 °C for 24 h (two 12-h exchanges), lyophilized, and stored for further use. Conjugate composition (enzyme and polymer/oligoamine content) and stoichiometry were determined using CD and FTIR spectroscopy (Supplementary Figure S2). Protein concentration was quantified using a Calcar formula based on amide I and amide II band intensities in the FTIR spectra (0.0056 absorbance units and 0.00346 absorbance units corresponding to 1 mg/mL protein for amide I and amide II, respectively). Polymer content was determined from the intensity of the 1000–1030 cm^−1^ band in the FTIR spectra. CD spectroscopy provided an additional measure of enzyme concentration within the conjugates.

PEC were formulated in 0.01 M PBS (pH 7.4) at 37 °C. L-ASNase conjugates (5 mg/mL in PBS) and heparin (10 mg/mL in PBS) were combined, vigorously mixed, and subjected to two 10-min sonication cycles. FTIR spectra of enzymes, conjugates, and PECs were acquired using either a MICRAN-3 FTIR microscope (Simex, Novosibirsk, Russia) or a Bruker Tensor 27 spectrometer (Bruker Optics, Ettlingen, Germany) equipped with a liquid-nitrogen-cooled MCT detector. Particle size and zeta potential were measured by dynamic light scattering (DLS) using a Zetasizer Nano S (Malvern Instruments, Worcestershire, UK). Circular dichroism (CD) spectroscopy with a Jasco J-815 CD spectrometer (JASCO, Tokyo, Japan) determined the enzyme concentration. Finally, the morphology and size of the particles were visualized and compared using atomic force microscopy (AFM) with a NTEGRA II AFM microscope (NT-MDT Spectrum Instruments, Moscow, Russia).

### 3.4. Determination of L-ASNase Catalytic Activity

The kinetic parameters, specifically *K*_M_ and V_max_, were ascertained in accordance with standard procedures by employing nonlinear regression analysis on experimental data acquired through the use of the fluorimetric technique and the Asp-AMC substrate.

#### 3.4.1. Fluorimetric Assay

L-ASNase activity was assessed by adding the enzyme formulation (0.1–100 µg/mL final concentration) to a 1 mM solution of Asp-AMC in 0.01 M PBS (pH 7.4) or in bovine serum, or whole human blood mixed with PBS. Following mixture, the kinetic curves were monitored fluorometrically (excitation, 360 nm; emission, 460 nm). Enzyme activity (U/mg) was calculated from the initial linear portion of the kinetic curves (0–10 min), using a standard EcA preparation as a reference. Product accumulation was also quantified at various time points (2, 4, 6, 12, and 24 h). The limit of detection (LOD) and limit of quantification (LOQs) were defined as 3 and 10 times the standard deviation (SD), respectively.

#### 3.4.2. Fourier-Transform Infrared Spectroscopy

FTIR spectra of asparagine (Asn, 20–40 mM) in PBS, bovine serum, or human whole blood mixed with PBS were acquired every minute at a resolution of 2 cm^−1^, using a Bruker Tensor 27 spectrometer (Bruker, Ettlingen, Germany) equipped with a liquid-nitrogen-cooled MCT detector and a thermostat (Huber, Raleigh, NC, USA). Spectra were obtained by averaging 70 scans. Typically, enzyme (or conjugate) in 0.01 M PBS (pH 7.4) was added to the Asn solution (in the same buffer) to achieve a final Asn concentration of 20–40 mM and an enzyme concentration of 0.001–0.1 mg/mL (adjusted based on specific activity). Reactions were performed at 37 °C under substrate-saturating conditions to ensure maximum reaction velocity.

To construct kinetic curves, we used a band 1680 cm^−1^ corresponding to C(=O)NH_2_ of Asn (Supplementary Figure S4). The FTIR spectra were recorded in 5-min increments for 30–60 min. By tracking the decrease in this band’s intensity, we converted ATR units linearly into concentration units. Using the material balance equation ([Asp] = C_0_(Asn) − [Asn]), we then constructed the Asp product accumulation curves. By analyzing the tangent of the angle of inclination in the initial section, we determined Vmax and subsequently calculated k_cat_.

#### 3.4.3. Circular Dichroism (CD) Spectroscopy

CD measurements were conducted on a Jasco J-815 CD spectrometer (JASCO, Tokyo, Japan) with a temperature-controlled cell. Enzyme (or conjugate) in 0.01 M PBS (pH 7.4) was added to a 10 mM Asn solution (prepared in the same buffer) to a final enzyme concentration of 0.005–0.1 mg/mL (depending on specific activity). Reactions were performed at 37 °C, and kinetic data were obtained by monitoring changes in ellipticity at 210 nm (Supplementary Figure S7). Michaelis–Menten parameters were derived from the resulting kinetic curves.

### 3.5. Fluorescent Determination of Binding of Bovine Serum Proteins to L-ASNase and Its Conjugate with Polymers

The preparation of the samples started with the covalent attachment of eosin (1 µg/mL of eosin–isothiocyanate) to the ASPase (EwA) (1 mg/mL)—with EwA–eosin formation. Then, EwA–eosin was modified to generate the EwA-sp conjugate or was functionalized with PEI-g-PEG to obtain the EwA-PEI-g-PEG conjugate, as described in Section 3.3. Next, the fluorescence measurement stage involved the dilution of blood serum samples by preparing different concentrations with phosphate-buffered saline (PBS) at pH 7.4. A fixed volume of each L-ASNase solution was added to each of the diluted serum samples. These mixtures were then incubated at 37 °C to allow for proper interaction between the conjugates and the serum components. Subsequently, fluorescence intensity was measured using the SpectraMax M5 device (Sunnyvale, PA, USA) in the Costar black/clear-bottom plate (96 wells).

### 3.6. Determination of Ex Vivo Asparaginase Activity in Model Mice

#### 3.6.1. Animal Studies

The study was conducted in compliance with the OECD Principles on Good Laboratory Practice (OECD GLP, ENV/MC/CHEM(98)17) and the Russian Federation’s Interstate Standard on Laboratory Practice (GOST 33044-2014). All experimental procedures were executed in accordance with the formally approved Research Plan and Standard Operating Procedures (SOPs) of the CBI/LBI (Laboratory of Biomedical Investigations) of the Institute of Biomedical Chemistry of the Russian Academy of Sciences (IBH RAS).

Animal welfare was a primary concern. The CBI/LBI IBH RAS holds a valid Veterinary Certificate, No. 0045092, issued on 29 December 2023. All animal-based experiments were conducted in accordance with the Institute’s Program for the Care and Use of Laboratory Animals.

All animal procedures were meticulously documented in the research plan, SOPs, and the Application Protocol for the Use of Animals, which were submitted to the Institute’s Bioethical Commission for review and approval. The Commission’s assessment ensured that all experimental protocols complied with applicable regulatory documents.

#### 3.6.2. Activity Assays

A screening study was conducted to assess the efficacy of experimental L-ASNase formulations in vivo, using mice with L5178Y Fisher lymphadenosis model tumors to develop a highly potent L-asparaginase medication for the treatment of leukemia in oncohematology with enhanced activity and reduced toxicity. The model was established by injecting the L5178Y mouse lymphoma cell line into DBA/2 mice intraperitoneally at a concentration of 1 × 10^6^ cells per animal. The animals were divided into groups of 6 individuals. The test objects were injected into animals intraperitoneally 24 h after inoculation of the cell line for 10 days (10-fold). The volume of administration was calculated based on the concentration of mg/kg, taking into account individual body weight values. The dose was administered to each animal according to the schedule of procedures and the dose-administration sheet. The test subjects received either L-ASNase at a dose of 2000 U/kg or a vehicle solution for the control group, which was administered intraperitoneally to the animals for 10 consecutive days, with injections occurring every 24 h. Blood samples were collected at specific time points during the study to determine asparaginase activity using fluorimetric methods. Additionally, clinical signs indicative of health status and body weight changes were monitored in the experimental animals. On the 60th day following the inoculation of the cell line, or in the event of a critical condition occurring earlier, the animals underwent necropsy, which included a macroscopic examination of their organs, as well as the weighing and fixation of their mesenteric lymph nodes and spleen.

To conduct the experiment, a known amount of enzyme (0.012 mg of EwA, equivalent to 3.4 units in PBS and 3.2 units in serum based on previous calibrations) was added to a homogeneous rat liver homogenate (100 mg + 400 μL PBS). After that, IR spectra of 50 mM asparagine were recorded during hydrolysis using a 2 μL aliquot of EwA extracted from 450 μL of liver homogenate (derived from 100 mg tissue) at pH 7.4 and T = 37 °C. The amount of enzyme present was then determined and compared to the amount initially added.

## 4. Conclusions

Acute lymphoblastic leukemia (ALL) is the most common childhood cancer, and L-asparaginase (L-ASNase) is a crucial enzyme in its treatment. L-ASNase hydrolyzes asparagine, leading to apoptosis in leukemic cells through asparagine depletion, cell cycle arrest, and receptor-mediated effects. However, the efficacy of L-ASNase is influenced by its interactions with cells, immunogenicity, stability, and pharmacokinetics. Conjugation with polycations or encapsulation in polymeric nanoparticles are strategies employed to improve these characteristics. A major limitation of current analytical methods for L-ASNase activity is their difficulty in handling complex biological samples like whole blood and serum.

This study addresses this challenge by introducing a robust and highly sensitive method for assessing L-ASNase activity both in vitro and ex vivo, even in opaque media. By combining fluorometric analysis with FTIR spectroscopy, we gain a comprehensive understanding of L-ASNase catalysis at the molecular level in various environments. This dual approach enables comparison of kinetic parameters and stability of various L-ASNase (intracellular RrA, and periplasmic EcA and EwA) and formulations, including polyamine conjugates and PECs.

Our findings demonstrate that the observed activity of native EwA decreases in complex biological media, likely due to protein–protein interactions and proteolytic degradation by plasma enzymes. Modified EwA maintains or enhances its activity, highlighting the protective effects of PEI-g-PEG modification and the advantages of conjugates and PECs delivery. Similarly, PEI-g-PEG modification improves RrA activity in the presence of blood components. Highly sensitive fluorescence-based L-asparaginase activity assay, with detection limits of 0.001–0.005 U/mL (achieved through overnight product accumulation), offers significant advantages for pharmacokinetic studies, enabling detection of residual activity at levels and time points unattainable with standard methods (0.01–0.05 U/mL). This enhanced sensitivity is crucial for optimizing dosing regimens and understanding long-term efficacy. Its applicability extends to in vivo distribution studies, as demonstrated by quantifying L-asparaginase activity in liver homogenates, revealing partial enzyme inactivation in this complex biological environment and highlighting the limitations of extrapolating in vitro activity to in vivo situations. This method contributes significantly to anti-leukemia drug development and personalized medicine by enabling detailed activity profiling.

Therefore, we suggest a strategy for L-asparaginase activity determination tailored to specific research needs. We suggest optimal choice when, for example, one needs a high-throughput assay, or a high-sensitivity assay to determine a minimal residual activity in blood sample, or when the media is optically non-transparent, or other difficult cases. Fluorescence spectroscopy with Asp-AMC substrate is optimal for high-throughput screening in biological fluids, while FTIR spectroscopy is recommended for the molecular detailed of the enzyme–substrate interaction studies. For precise assessment of the residual activities in pharmacokinetic analyses, particularly with low detection limits, the Asp-AMC fluorescence method is superior, necessitating product accumulation over 12–24 h for enhanced sensitivity, and requiring separation of serum from blood cells when using whole blood. Both fluorescent and FTIR methods are applicable for evaluating complex L-ASNase formulations (e.g., conjugated, micellar, or liposomal), with accurate quantification substrate and product absorption coefficients and quantum yields across various aqueous-buffer and biological media, thus supporting the medical applications.

## Figures and Tables

**Table 1:** Physicochemical parameters of the studied native L-ASNases. L-ASNase activity parameters’ specific activity (U/mg) and *K*_M_ values were determined by CD spectroscopy using Asn as a substrate (*n* = 3, data presented as a mean ± SD), PBS (0.01 M, pH 7.4) 37 °C. [S]_0_ = 10 mM.

Enzyme	Type	Quaternary Structure	MW (Monomer), kDa	Number of Amino acid Residues	pI	pH-Optimum	T-Optimum, °C	Specific Activity *, U/mg	*K*_M_, mM	L-Glutaminase Activity, % of L-Asparaginase Activity
RrA	Ia	Tetramer/dimer	18	172	5.0–5.2	9.0–9.3	50–55	60 ± 6	4 ± 0.3	0.05
EcA	II	Tetramer	37	348	4.6–5.5	7–8.5	45–50	330 ± 20	0.020 ± 0.003	3
EwA	II	Tetramer	34	330	7.5–8	8.5–9.0	35–40	370 ± 15	0.030 ± 0.005	3

* L-ASNase activity may vary between different batches, typically ranging within a broader range between 350 and 500 U/mg.

**Table 2:** Physicochemical properties and kinetic parameters of native EwA, EcA, and RrA enzymes; its conjugated formulations; and PEC formulations with heparin. L-ASNase activity parameters were determined by fluorescence spectroscopy, using Asp-AMC as a substrate at 37 °C in bovine serum (pH 7.4). [S]_0_ = 1 mM.

L-ASNase Formulations	Molar Ratio of Components **	Zeta Potential of ASP Formulation mV	*K*_M_, µM	Specific Activity in PBS, U/mg	Specific Activity in 50% Serum, U/mg	Specific Activity in 100% Serum, U/mg
*EwA*	-	−1 ± 0.2	30 ± 5	280 ± 15	230 ± 20	270 ± 10
*EwA–spermine (EwA-sp)*	1:80	+4 ± 0.7		500 ± 40	240 ± 10	210 ± 15
*EwA-PEI-g-PEG*	1:1.5	+9 ± 2		300 ± 20	285 ± 30	350 ± 20
*EwA-PEI-g-PEG + heparin* → PEC *	1:1.5:8	−6 ± 1		340 ± 25	345 ± 20	415 ± 40
*RrA*	-	+3 ± 0.5	4000 ± 300	35 ± 3	40 ± 2	42 ± 3
*RrA-PEI-g-PEG*	1:1.5	+10 ± 1		38 ± 2	68 ± 4	54 ± 5
*EcA*	-	−3.5 ± 0.5	20 ± 3	380 ± 10	195 ± 10	140 ± 7

* EwA conjugates are covalent; the complexes with heparin are electrostatic (PEC), non-covalent. ** The ratio is given based on the monomer

**Table 3:** The limit of detection and limit of quantification of L-ASNases activity for Asp-AMC hydrolysis (1 mM). λexci = 360 nm. λemi = 460 nm. pH = 7.4. T = 37 °C.

The Limit of Detection (Limit of Quantification) of L-ASNase Activity, U/L			
Determination based on	in PBS	in 50% serum	in 100% serum
The initial section of the kinetic curve	170 (570)	150 (490)	40 (130)
Product accumulation in 12 h	10 (33)	21 (70)	26 (86)

**Table 4:** An example of determining the asparaginase activity of EwA and EwA-sp preparations in the blood serum of mice taken 20 days after 10 days of intraperitoneal daily administration of 4000 U/kg of drugs (total 20th day). L-ASNases’ activity was determined by fluorescence for Asp-AMC hydrolysis (1 mM). λexci = 360 nm. λemi = 460 nm. pH = 7.4. T = 37 °C. The calibrations shown in Figure 4 were used. Blood serum was used without dilution with buffer.

Group		Control	EwA (10 Days: 4000 U/kg Daily)	EwA-sp (10 Days: 4000 U/kg Daily)
**Initial activity, U/mL**		0	2.1 ± 0.4	2.3 ± 0.5
**Activity after 10 days of treatment, U/mL** (**accumulation of enzyme in 10 days**)		0	4.9 ± 0.4	10 ± 1
Residual activity 20 days after the 10 days enzyme treatment, U/mL *{without taking into account the background}*	n = 1	0.57	0.62	0.49
	n = 2	0.58	0.60	0.70
	n = 3	0.57	0.58	0.63
	n = 4	0.55	0.58	0.69
	n = 5	0.57	0.59	0.66
	n = 6	0.55	0.61	0.70
	**Average**	**0.56**	**0.60**	**0.65**
	**SD**	0.01	0.02	0.08
Residual activity 20 days after the 10 days enzyme treatment, U/mL *{minus background}*	n = 1	0.01	0.06	−0.07
	n = 2	0.02	0.03	0.14
	n = 3	0.00	0.01	0.07
	n = 4	−0.02	0.01	0.12
	n = 5	0.01	0.03	0.10
	n = 6	−0.01	0.05	0.14
	**Average**	**0.00**	**0.03**	**0.08**
	**SD**	0.01	0.02	0.08

**Table 5:** Experimental possibilities of various methods for determining L-asparaginase activity.

Possibilities of the Method	Fluorimetry with Asp-AMC [45,46]	FTIR Spectroscopy [37]	CD Spectroscopy [24,44]	pH-Stat Titration [47]	Conductometry [44]	Nesslerization and Spectrophotometric Techniques Based on Coupled Enzymatic Reaction [32,48,49]
Specific activity	+	+	+	+	+	+
Determination of V_max_	+	+	+	+	+	–
Determination of *K*_M_	+	For type I enzymes	+	+	+	+/–
Analysis of biological media (blood serum, whole blood, and organs)	+	+	–	–	–	–
Methodical simplicity	+	–	+/–	–	–	+/–
Analysis of conjugates and asparaginase formulations	+	+	+	+/–	–	–
High sensitivity	+	–	+/–	–	–	+/–

## Data Availability

The data presented in this study are available in the main text and Supplementary Materials.

## Supplementary Materials

The following supporting information can be downloaded at https://www.mdpi.com/article/10.3390/ijms26115227/s1.

## Abbreviations

The following abbreviations are used in this manuscript:

ALL	Acute lymphoblastic leukemia
Asp-AMC	L-aspartic acid β-(7-amido-4-methylcoumarin)
AMC	7-amino-4-methylcoumarin
CD	Circular dichroism
FTIR	Fourier-transform infrared
L-ASNase	L-asparaginase
LOD	Limit of detection
LOQ	Limit of quantification
PEC	Polyelectrolyte complex
PEG	Polyethylene glycol
PEI	Polyethyleneimine
