# Supplementary material for: A Novel Approach for the Activity Assessment of L-Asparaginase Formulations When Dealing with Complex Biological Samples
Source: Int J Mol Sci. 2025 May 29;26(11):5227. doi: 10.3390/ijms26115227 (PMC12154015; doi:10.3390/ijms26115227)
Supplement: Supplementary file 1 [file ijms-26-05227-s001.zip › ijms-3648412-supplementary.pdf]

Table S1. The main analytically significant absorption bands in the spectra of L-asparagine and L-aspartic acid.

Compound	Peak position, cm^{-1}	Assignment
L-aspartic acid and L-asparagine	1580	ν_s COO-
	1420	ν_{as} COOH
	1390	ν_{as} COO^-
	1150	ν $C\text{-}NH_3^+$
L-asparagine	1680	ν C=O in $C(O)NH_2$
	1610	ν_s COO-

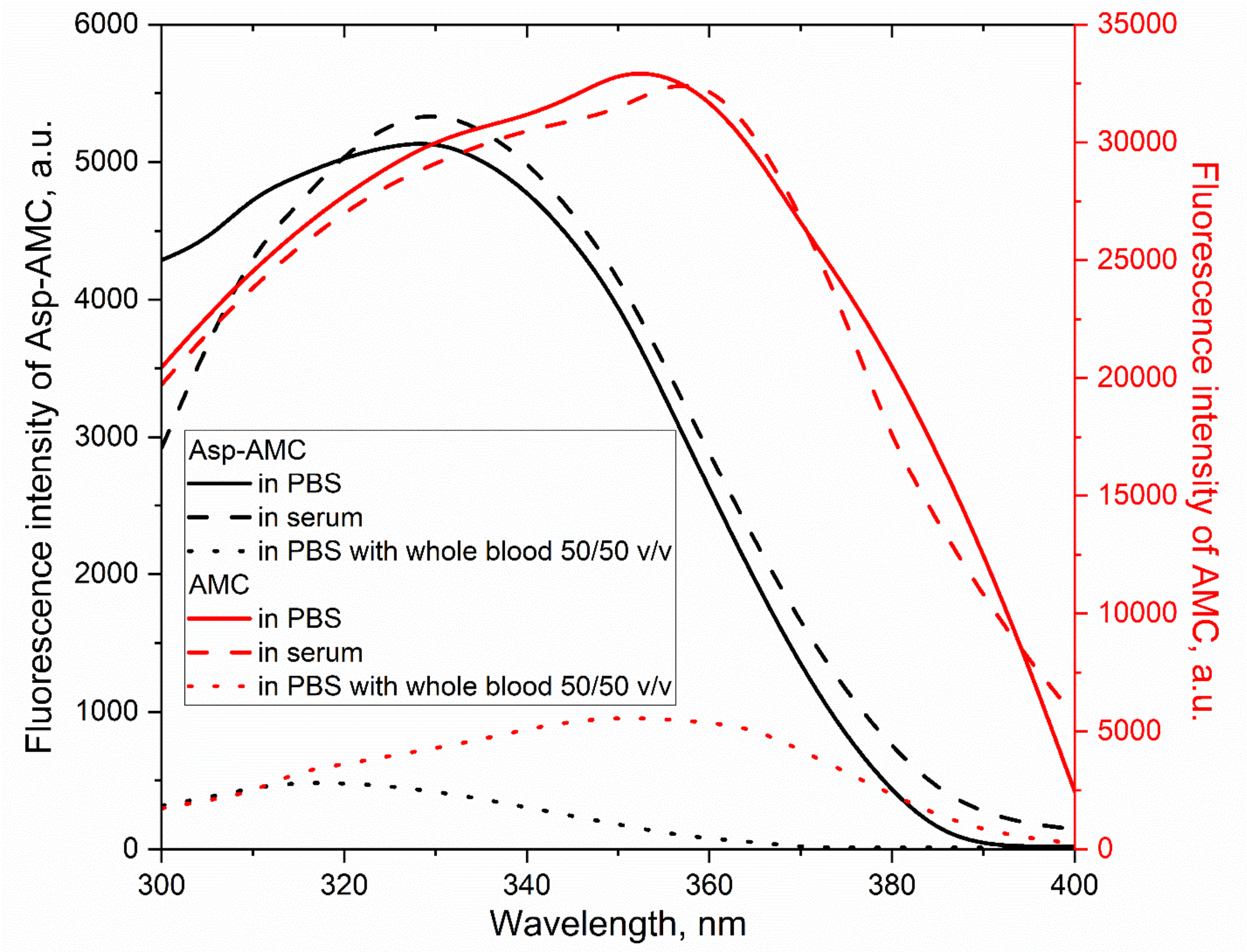

Figure S1. Fluorescence excitation spectra of 5 µM AMC fluorescent product and Asp-AMC substrate for L-ASNase in PBS buffer, bovine blood serum, and whole blood diluted with PBS (50/50 v/v). λemi = 460 nm. pH = 7.4. T = 37 °C.

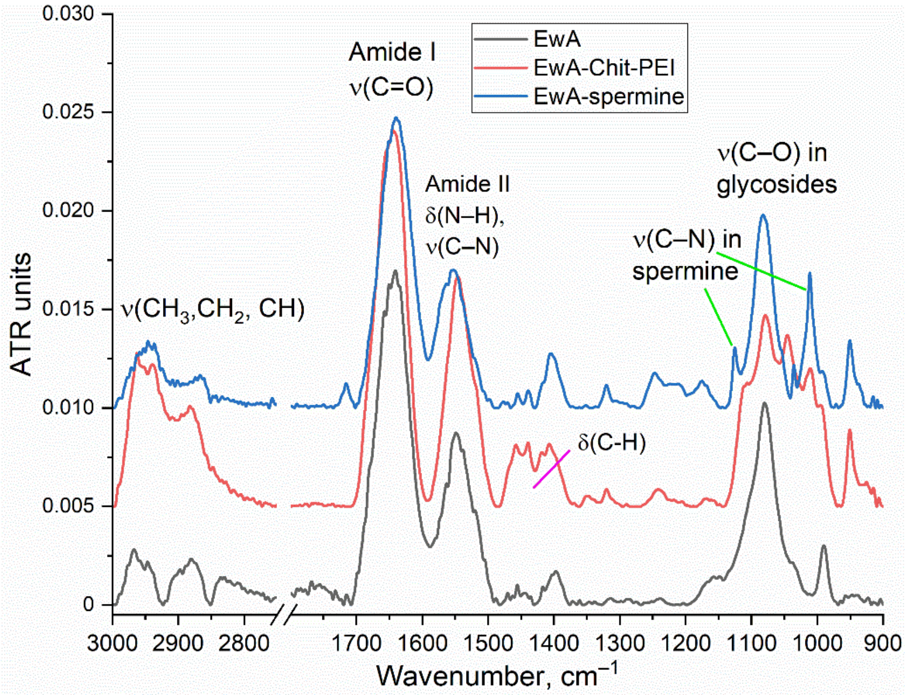

Figure S2. FTIR spectra of native protein *Erwinia carotovora* L-ASNase (EwA) and EwA conjugated with PEG-PEI, spermine. PBS (0.01 M, pH 7.4). T = 22 °C.

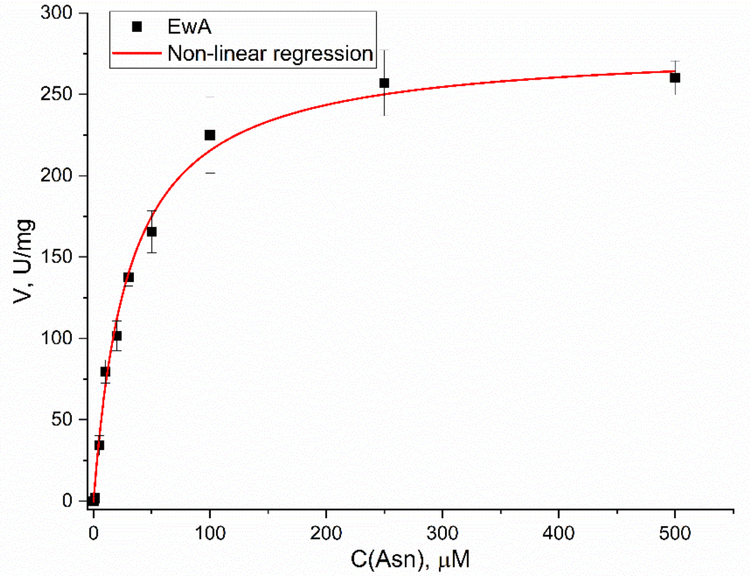

(a)

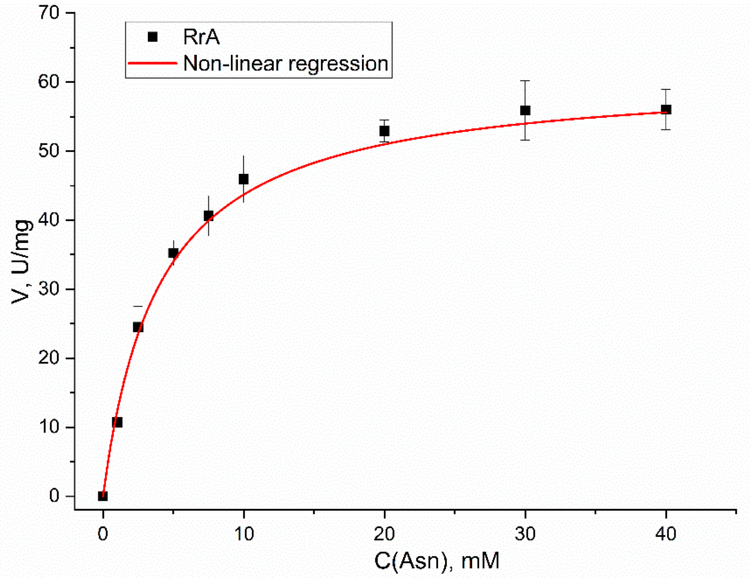

(b)

Figure S3. Examples of Michaelis curves of substrate specificity of (a) EwA and (b) RrA. L-ASNase activity parameters were determined by fluorescence spectroscopy using Asp-AMC as a substrate at 37 °C in PBS (pH 7.4).

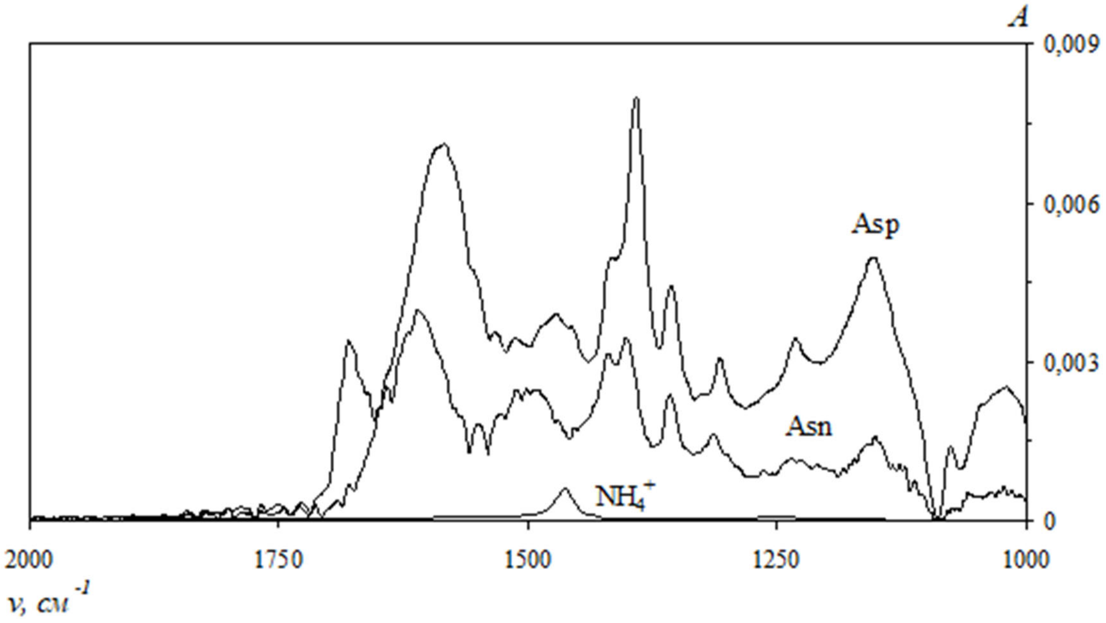

(a)

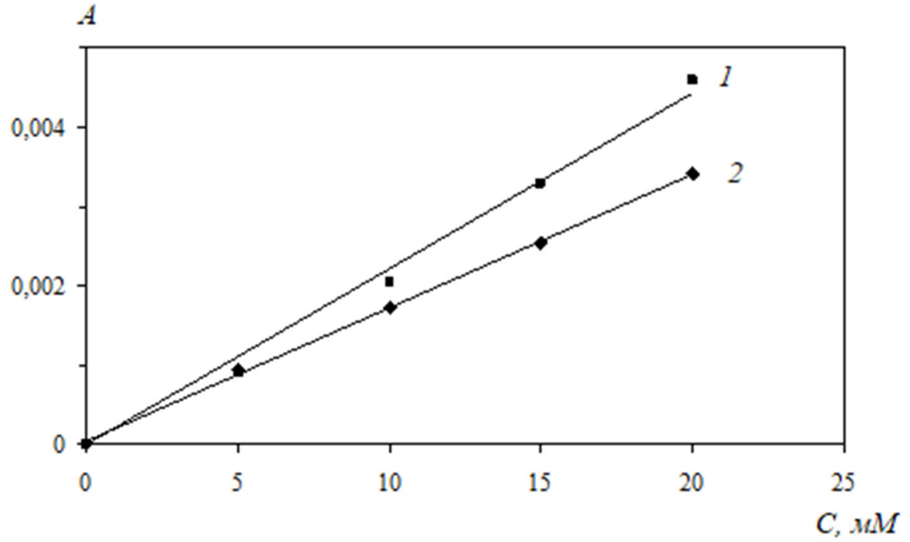

(b)

Figure S4. (a) FTIR spectra of L-aspartic acid, L-asparagine and ammonium ion. [L-Asn] = [L-Asp] = [NH4+] = 20 mM, (15 mM sodium phosphate buffer, pH 7.4, 37 °C). (b) Calibration dependences of A(1680 cm^{-1}) on the concentration of L-asparagine (1) and A(1580 cm^{-1}) on the concentration of L-aspartic acid. [L-Asn], [L-Asp] = 1 – 20 mM, (15 mM sodium phosphate buffer, pH 7.5, 37oC)

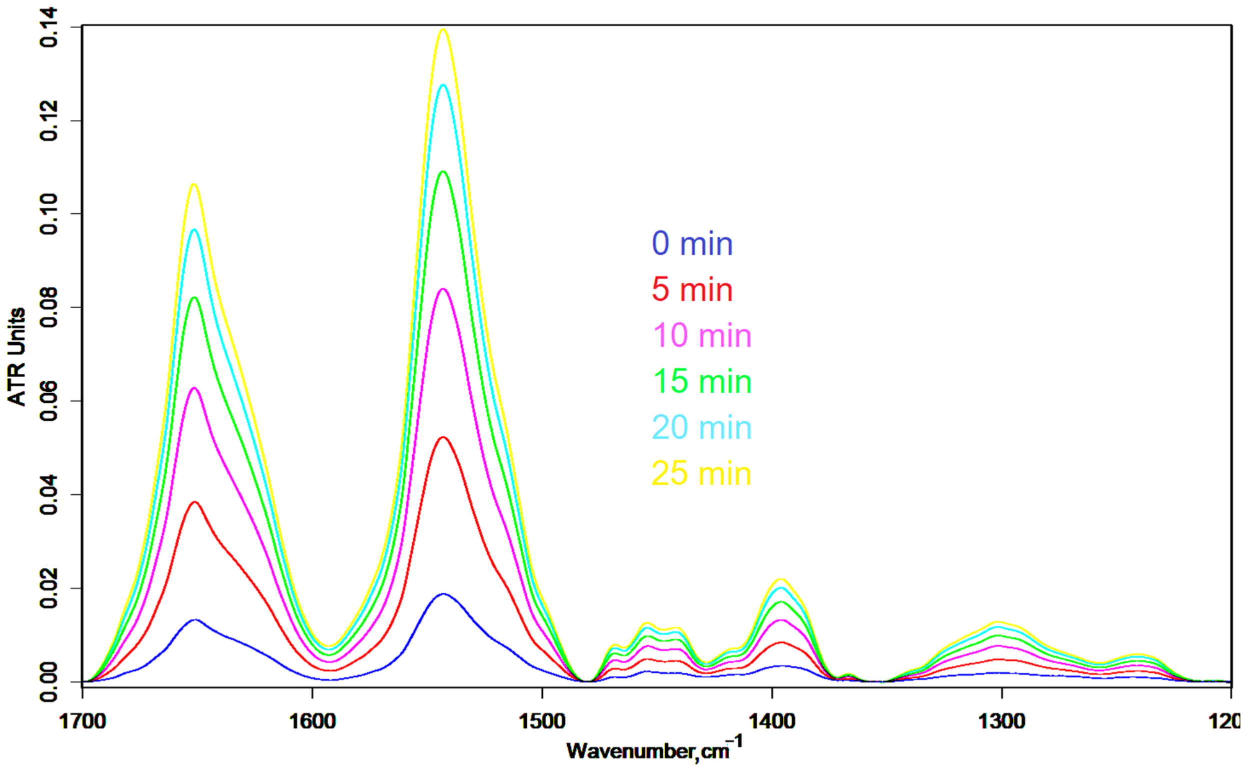

Figure S5. FTIR spectra of 5% blood cells +95% PBS v/v + EwA (300 nM). The data was recorded in 5-minute increments, serving as a backdrop for the process of cellular deposition. PBS (0.01 M, pH 7.4). T = 37 °C. The acquisition time is 1 minute/

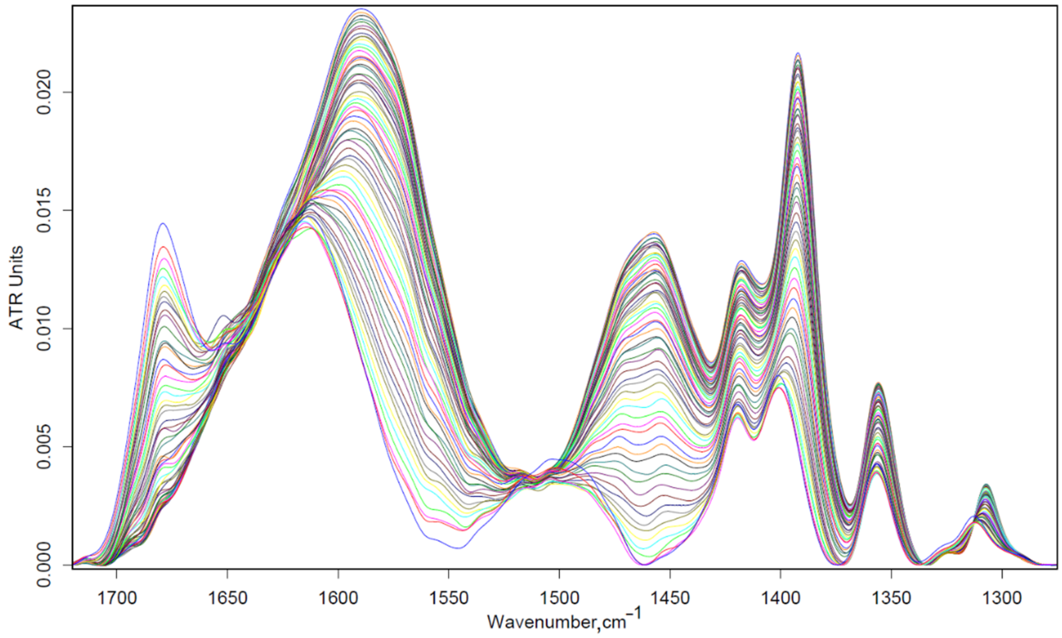

(a)

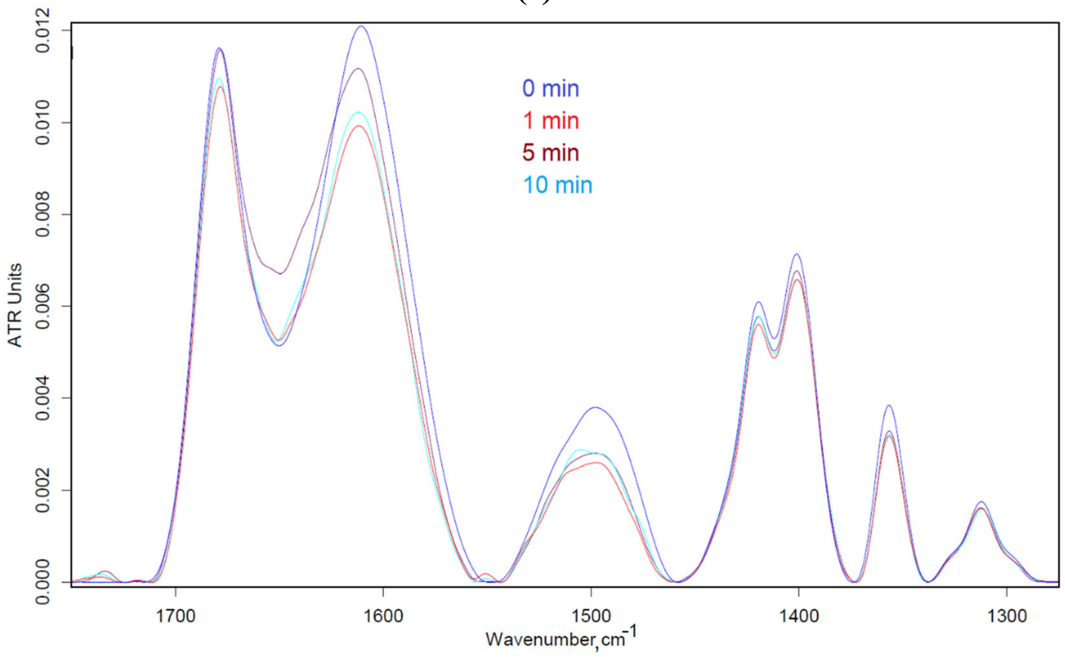

(b)

Figure S6. FTIR spectra of asparagine (30 mM) during catalytic hydrolysis by (a) 0.1 U of EwA with a time step of 10 seconds, (b) EwA enzyme in the blood serum when the activity is less than the limit of detection. The contribution of blood proteins is taken into account as the background. PBS (0.01 M, pH 7.4). T = 37 °C.

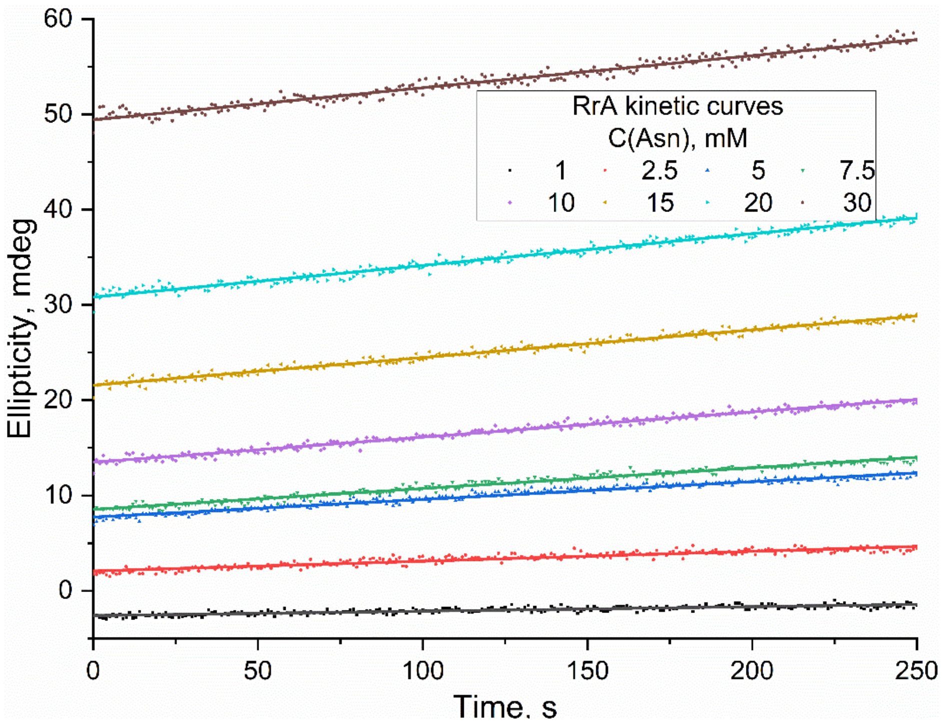

Figure S7. Examples of kinetic curves of Asn hydrolysis by RrA recorded using the CD spectroscopy method. Conditions: 10 mM phosphate buffer, pH 7.4, temperature 37°C, wavelength 210 nm.
